# Sphingosine Kinase 1 Signaling in Breast Cancer: A Potential Target to Tackle Breast Cancer Stem Cells

**DOI:** 10.3389/fmolb.2021.748470

**Published:** 2021-11-08

**Authors:** Ling-Wei Hii, Felicia Fei-Lei Chung, Chun-Wai Mai, Pei Yuen Ng, Chee-Onn Leong

**Affiliations:** ^1^ Department of Life Sciences, School of Pharmacy, International Medical University, Kuala Lumpur, Malaysia; ^2^ Center for Cancer and Stem Cell Research, Institute for Research, Development and Innovation (IRDI), International Medical University, Kuala Lumpur, Malaysia; ^3^ Department of Medical Sciences, School of Medical and Life Sciences, Sunway University, Bandar Sunway, Malaysia; ^4^ State Key Laboratory of Oncogenes and Related Genes, School of Medicine, Renji-Med X Clinical Stem Cell Research Center, Ren Ji Hospital, Shanghai Jiao Tong University, Shanghai, China; ^5^ Drug and Herbal Research Centre, Faculty of Pharmacy, Universiti Kebangsaan Malaysia, Kuala Lumpur, Malaysia

**Keywords:** sphingosine kinase, sphingosine-1-phosphate, sphingosine-1-phosphate receptor, breast cancer, cancer stem cells, sphingosine kinase inhibitor

## Abstract

Sphingosine kinases (SPHKs) are conserved lipid enzymes that catalyze the formation of sphingosine-1-phosphate (S1P) through ATP-dependent phosphorylation of sphingosine. Two distinct SPHK isoforms, namely SPHK1 and SPHK2, have been identified to date, and the former has been implicated for its oncogenic roles in cancer development and progression. While SPHK1 signaling axis has been extensively studied in non-stem breast cancer cells, recent evidence has emerged to suggest a role of SPHK1 in regulating cancer stem cells (CSCs). With the clinical implications of CSCs in disease relapse and metastasis, it is believed that therapeutic approaches that can eradicate both non-stem cancer cells and CSCs could be a key to cancer cure. In this review, we first explore the oncogenic functions of sphingosine kinase 1 in human cancers and summarize current research findings of SPHK1 signaling with a focus on breast cancer. We also discuss the therapeutic potentials and perspectives of targeting SPHK1 signaling in breast cancer and cancer stem cells. We aim to offer new insights and inspire future studies looking further into the regulatory functions of SPHK1 in CSC-driven tumorigenesis, uncovering novel therapeutic avenues of using SPHK1-targeted therapy in the treatment of CSC-enriched refractory cancers.

## Introduction

**FIGURE 1:**
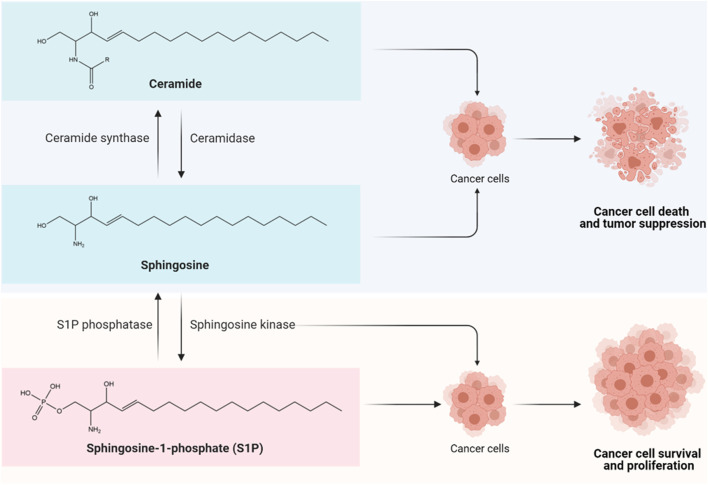
The sphingolipid rheostat. The sphingolipid rheostat describes the interconversion between ceramide, sphingosine, and sphingosine-1-phosphate (S1P) for cell fate determination. Ceramide is composed of a long-chain sphingosine base containing 18 carbons and an amide-linked fatty acyl chain which can have 14 to 26 carbons in length (Ogretmen, 2018). Ceramide is deacylated by ceramidase to form sphingosine, followed by phosphorylation by sphingosine kinase (SPHK) to produce S1P. The synthesis and accumulation of ceramide and/or sphingosine can induce cancer cell death and tumor suppression through apoptosis, necroptosis, autophagy, and cell cycle arrest (Hannun and Obeid, 2018; Ogretmen, 2018); while the biosynthesis of S1P that is driven by SPHKs, particularly SPHK1, appears to exert pro-survival and anti-apoptotic effects in cancer cells via S1P receptor (S1PR)-dependent or -independent signaling pathways, leading to enhanced cancer cell growth, therapy resistance, tumor invasion and metastasis (Hannun and Obeid, 2018; Ogretmen, 2018).

Sphingolipids are one of the main classes of bioactive lipid molecules found in eukaryotic cells. Historically, sphingolipids were first isolated by Thudichum from brain tissue in the late 19th century, at which time the term “sphingosin” was coined, likening sphingolipids to a mythical creature of Greek called Sphinx, owing to the enigmas presented by these lipids upon discovery (Thudichum, 1884). While sphingolipids were initially regarded as mere structural components of eukaryotic cell membranes, compelling evidence has described the vast complexity of sphingolipid metabolism, leading to the discovery of additional sphingolipid functions as second messengers and as bioactive signaling molecules. Among them, the main sphingolipid molecules that have always received researchers’ attention include ceramide, sphingosine, and sphingosine-1-phosphate (S1P). These bioactive sphingolipids regulate various biological processes in cells, and the ceramide-sphingosine-S1P rheostat has been implicated as an important mechanism balancing the growth, survival, and cell fate of mammalian cells (Hannun and Obeid, 2018). In brief, the sphingolipid rheostat is a concept proposed to describe how interconversions between pro-apoptotic ceramides and pro-survival S1P attenuate cell fate (Figure 1). In the sphingolipid cycle, ceramide is deacylated into sphingosine by ceramidase, and sphingosine can be further phosphorylated to form S1P by sphingosine kinase (SPHK); in turn, S1P can either be irreversibly degraded by S1P lyase, or dephosphorylated to sphingosine via S1P phosphatase, followed by re-acylation back to ceramide by ceramide synthase (Hannun and Obeid, 2018; Ogretmen, 2018). Such interconversions are rapid and mediated through compartment-specific processes. The distinct roles and downstream targets of these bioactive sphingolipids are highly context- and cell type-dependent.

As accumulating evidence indicates that ceramide/sphingosine and S1P have opposing functions in oncogenesis, it is becoming increasingly clear that the dysregulation of the sphingolipid rheostat is a key event of cancer initiation. The production and accrual of ceramide and/or sphingosine in response to stress stimuli are known to induce cancer cell death and tumor suppression via apoptosis, necroptosis, autophagy, and cell cycle arrest (Hannun and Obeid, 2018; Ogretmen, 2018). In stark contrast, the biosynthesis of S1P that is driven by SPHKs, particularly SPHK1, appears to exert pro-survival and anti-apoptotic effects in cancer cells, by promoting cancer cell proliferation, therapy resistance, tumor invasion and metastasis via S1P receptor (S1PR)-dependent and/or -independent signaling pathways (Hannun and Obeid, 2018; Ogretmen, 2018). Furthermore, recent studies have identified a role of SPHK1 in mediating survival of breast cancer stem cells (CSCs) (Hirata et al., 2014; Wang et al., 2016; Hii et al., 2020). It is believed that the multiple signaling nodes involved in this rheostat, including the bioactive sphingolipids, enzymes, and receptors, are potential targets for theragnostic advancement in CSC-driven refractory breast cancers.

In this review, we first explore the key functions of sphingosine kinase 1 in human cancers, followed by a critical review of current findings on SPHK1 signaling, particularly in breast cancer. We also discuss current therapeutics and perspectives of targeting SPHK1 signaling in breast cancer and cancer stem cells. Taken together, this review aims to offer new insights and inspire future studies into the regulatory functions of SPHK1 in CSC-driven breast tumorigenesis. It is anticipated that a better understanding in this aspect of CSC biology will uncover novel therapeutic avenues, adding relevant targeted strategies for the treatment of CSC-enriched breast cancers.

## Sphingosine Kinase 1 as a Key Player of Oncogenesis

Sphingosine kinases (SPHKs) are evolutionary conserved lipid kinases that catalyze the generation of S1P from sphingosine through ATP-dependent phosphorylation. Five conserved regions, denoted from C1 to C5, have been found within SPHKs, whereby C1 to C3 domains and C4 to C5 domains are known to reside at N-termini and C-termini, respectively (Liu et al., 2000; Pitson, 2011). The C1 to C3 domains in SPHKs consist of the diacylglycerol (DAG) kinase catalytic region which is commonly presented in ceramide kinase and DAG kinases, while C4 represents a unique domain in SPHKs (Alemany et al., 2007). The presence of C4 domain has distinguished SPHKs from other lipid enzymes and granted them the exclusive capability of converting sphingosine into S1P, making SPHKs the sole generators of S1P (Yokota et al., 2004).

**FIGURE 2:**
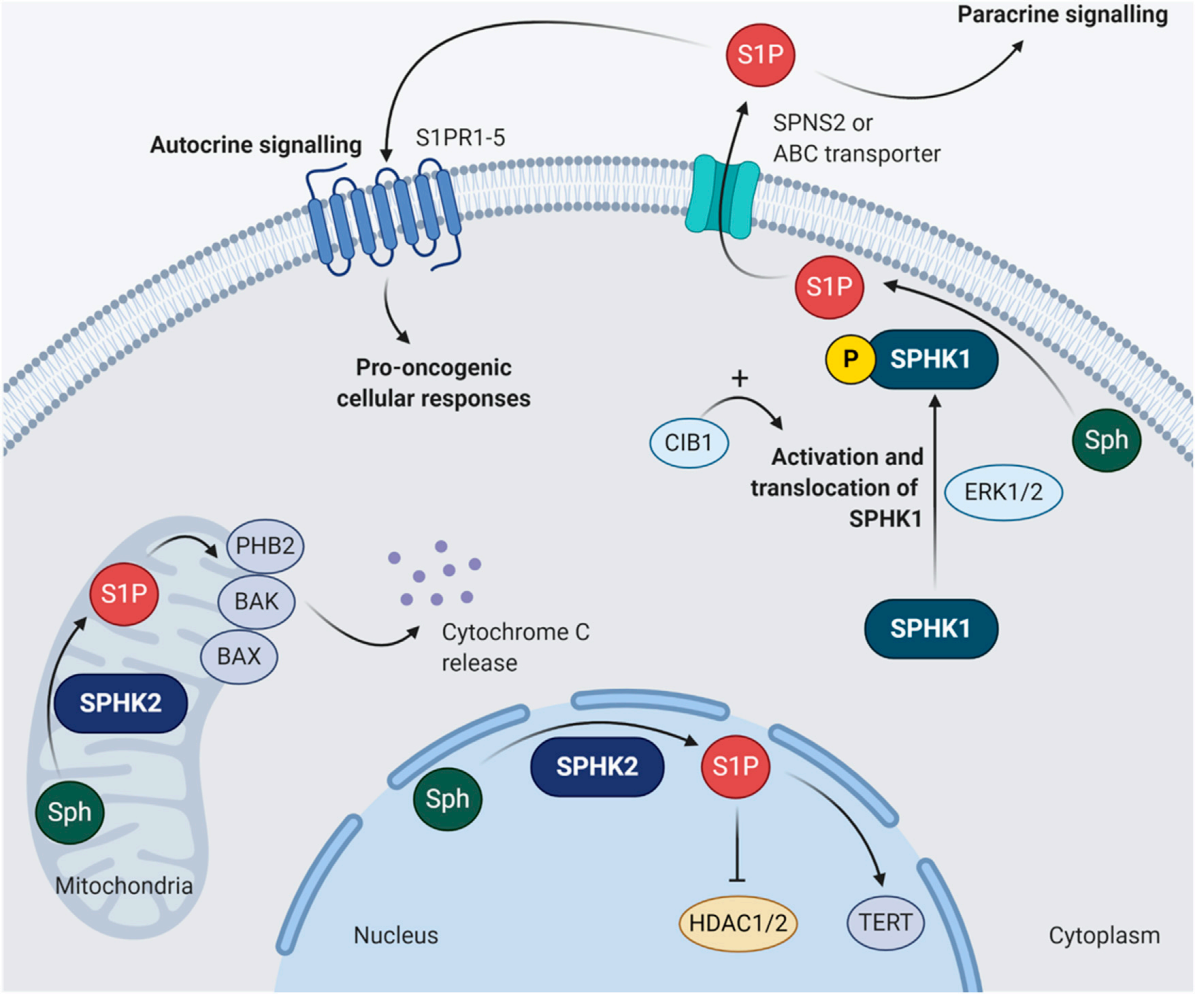
Subcellular localization of SPHK1 and SPHK2. SPHK1 is usually found in the cytoplasm. Once it is phosphorylated at serine 225 by ERK1/2, the activated SPHK1 undergoes translocation to the plasma membrane (Pitson et al., 2005). Such activation of SPHK1 activity can be further enhanced by CIB1 by exerting its function as Ca^2+^-myristoyl switch (Zhu et al., 2017). The SPHK1-generated S1P can be exported out of the cell by specific transporters involving SPNS2 or ABC transporters, followed by binding to S1PR1-5 and stimulate pro-oncogenic cellular responses via autocrine or paracrine signaling. As for SPHK2, it is mainly localized in the mitochondria and nucleus. Unlike SPHK1, the functions of SPHK2 in cancer appear to be much more complicated with contradictory findings. For instance, S1P produced in the mitochondria by SPHK2 can bind to PHB2, activate BAK/BAX and induce cytochrome c release (Strub et al., 2011; Chipuk et al., 2012); while the SPHK2-derived nuclear S1P can inhibit HDAC1/2 and induce epigenetic modulation of *CDKN1A* and *FOS*, which encode p21 and proto-oncogene FOS, respectively (Hait et al., 2009). However, the nuclear S1P generated by SPHK2 can also interact with TERT to stabilize telomerase, in turn inhibiting telomere damage and senescence (Panneer Selvam et al., 2015). ABC, ATP-binding cassette; BAK, Bcl-2 antagonist/killer; BAX, Bcl-2-associated X protein; CIB1, calcium and integrin binding protein 1; ERK1/2, extracellular signal-regulated kinases 1 and 2; HDAC1/2, histone deacetylases 1 and 2; PHB2, prohibitin 2; S1P, sphingosine-1-phosphate; S1PR1-5, sphingosine-1-phosphate receptors 1 to 5; Sph, sphingosine; SPHK1, sphingosine kinase 1; SPHK2, sphingosine kinase 2; SPNS2, protein spinster homolog 2; TERT, telomerase reverse transcriptase.

Up to now, there are two main isoforms of SPHKs been discovered in humans, namely SPHK1 and SPHK2. These two isoforms of SPHKs have different chromosomal locations and differ in their subcellular localization (Figure 2), developmental expression, and tissue distribution. SPHK1 is located on chromosome 17 and predominantly localized in the cytosol. SPHK2 is primarily nuclear and mitochondrial and is located on chromosome 19. Studies in mice have shown that peak Sphk1 expression can be detected at day 7 of mouse embryonic development and decreases thereafter. In contrast, Sphk2 expression is detected from day 11 and gradually increases up to day 17 (Liu et al., 2000). In adult mouse tissues, high levels of Sphk1 are found in the lung, spleen, kidney, and blood; while Sphk2 is mainly expressed in the brain, heart, kidney, and liver (Liu et al., 2000; Fukuda et al., 2003). These suggest that SPHK1 and SPHK2 may have different biological functions for creating different compartmental-specific S1P pools. At present, SPHKs have been reported to possess different roles in many physiological and pathological processes, including autoimmune diseases, immunosuppression, inflammation, infection, cancer, neurodevelopment, and degeneration (Kunkel et al., 2013; Pyne et al., 2016a; Hannun and Obeid, 2018). In the context of cancer, SPHK1 is the main isoform that is functionally associated with the hallmarks of cancer, which has been universally recognized as a key player of oncogenesis.

A meta-analysis of SPHK1 in human cancers has demonstrated significantly higher levels of SPHK1 in both benign and cancerous tissues as compared to normal tissues (Zhang et al., 2014). High expression of SPHK1 has been observed in various tumor types and is associated with poorer clinical prognosis and shorter overall survival in cancer patients (Zhang et al., 2014). Multiple independent studies have examined the effects of SPHK1 overexpression or depletion via RNA interference (RNAi) approaches in different cancer models and have uniformly established that SPHK1 possesses a role in promoting cancer cell growth and inhibiting apoptosis (Table 1). Overexpression of SPHK1 has been reported to enhance the Ras-dependent neoplastic transformation and induce the formation of tumors which are much larger, more vascularized and treatment resistant (Pyne et al., 2016b). Moreover, SPHK1 has been found to control cancer cell migration and modulate interaction of cancer cells with cancer-associated fibroblasts, contributing to tumor invasion and metastasis (Pyne et al., 2016b). Some have also shown that SPHK1 activity is responsible for the induction of inflammation and maintaining the Warburg effect and cell survival, which further enable the acquisition of cancer hallmarks in affected cells (Liang et al., 2013; Pyne and Pyne, 2013; Watson et al., 2013). It is worth mentioning that *Sphk1* knockout mice models have offered useful insights on the protective role of SPHK1 depletion in oncogenesis, with colon cancer as the pioneered context model. In view of the absence of SPHK1 mutations across various cancer types, it is suggested that tumors exhibit a dependency on hyperactivation of SPHK signaling that confers survival and growth advantages to cancer cells, a phenomenon known as “non-oncogenic addiction” (Vadas et al., 2008).

**TABLE 1:** Current findings of SPHK1 in human cancers.

Types of cancer	Findings	References
Breast cancer	Estrogen receptor (ER)-positive breast cancer	Taha et al. (2004), Sarkar et al. (2005), Taha et al. (2006), Long et al. (2010a), Watson et al. (2010), Datta et al. (2014), Hirata et al. (2014), Maiti et al. (2017), Wang et al. (2018), Acharya et al. (2019), Alshaker et al. (2019), Chen and Liu (2020), Hii et al. (2020)
	⁃ High expression of SPHK1, S1PR1 and S1PR3 were associated with poor prognosis in patients with ER-positive breast cancer	
	⁃ Knockdown of SPHK1 in ER-positive breast cancer reduced cell viability and induced cell cycle arrest and apoptosis involving caspase activation, cytochrome c release and Bax oligomerization	
	⁃ Knockdown of SPHK1 in ER-positive breast cancer reduced EGF-stimulated cell growth and improved sensitivity to doxorubicin	
	⁃ Estrogen treatment stimulated translocation of SPHK1 and S1PR3 into the ER-positive breast cancer cell nuclei	
	⁃ Oncogene tolerance between SPHK1 and HER2 was reported in ER-positive breast cancer cells with the involvement of p65 PAK1/ERK-1/2 signaling	
	⁃ SPHK1 and S1PR3 expression was higher in ER-positive breast cancer stem cells (CSCs) when compared to the respective non-CSCs	
	⁃ Ectopic expression of SPHK1 in ER-positive breast cancer cells promoted cell migration, induced epithelial-mesenchymal transition, and increased the stemness marker expression levels of SOX2, OCT4, NANOG, and ALDH1	
	⁃ Overexpression of SPHK1 promoted the growth and tumorigenicity of ER-positive breast CSCs via S1PR3/Notch signaling	
	Triple negative breast cancer (TNBC)	
	⁃ Compared to other subtypes, SPHK1 expression was significantly upregulated in TNBC patients and was associated with poor survival and response to doxorubicin	
	⁃ SPHK1 promoted metastasis by transcriptionally upregulating the expression of FSCN1 via NF-κB activation	
	⁃ Knockdown of SPHK1 suppressed EGF-mediated signaling of ERK/AKT/p38 MAPK in metastatic TNBC cells	
	⁃ Knockdown of SPHK1 in TNBC cells downregulated G3BP stress granule assembly factors (G3BP1 and G3BP2) that known to involve in NF-κB, RAS and Wnt signaling	
	⁃ Knockdown of SPHK1 in TNBC cells and CSCs inhibited the cell proliferation and induced apoptosis via STAT1/interferon-dependent mechanism	
	⁃ Inhibition of SPHK1 in TNBC cells and CSCs sensitized TNBCs to doxorubicin treatment	
	⁃ Compared to the corresponding non-CSCs, SPHK1 protein expression was higher in TNBC CSCs	
	⁃ Ectopic expression of SPHK1 promoted the mammosphere formation and survival of TNBC CSCs	
Lung cancer	⁃ Significant upregulation of SPHK1 was observed in non-small cell lung cancer (NSCLC) and associated with poor patient survival	Song et al. (2011), Zhu et al. (2015), Ma et al. (2021)
	⁃ Overexpression of SPHK1 increased the proliferation and migration of NSCLC via activation of PI3K/AKT/NF-κB and increased expression of Bcl-xl, c-IAP1, c-IAP2, TRAF1, Bcl-2, matrix metallopeptidase 2 and cyclin D1	
	⁃ Inhibition of SPHK1 potentiated NSCLC sensitivity to docetaxel or doxorubicin treatment	
Gastric cancer	⁃ Poor prognosis of patients with gastric cancer was found to be correlated with elevated expression of SPHK1	Li et al. (2009), Fuereder et al. (2011), Xiong et al. (2014)
	⁃ Knockdown of SPHK1 induced apoptosis in gastric cancer cells and upregulates Bim via AKT/FoxO3a pathway	
	⁃ Ectopic expression of SPHK1 prevented UV irradiation-induced cell death in gastric cancer cells	
	⁃ Inhibition of SPHK1 synergized doxorubicin sensitivity in gastric cancer cells	
Colon cancer	⁃ High SPHK1 expression correlated with advanced tumor stages in colon cancer patients	Kohno et al. (2006), Kawamori et al. (2009), Snider et al. (2009), Tan et al. (2014)
	⁃ In mouse models of colon carcinogenesis, colon tumor initiation and development occur at consistently much lower rates in *Sphk1* ^−/−^ mice	
	⁃ Inhibition of SPHK1 reduced the viability of colon cancer cells	
	⁃ SPHK1 regulates COX2/STAT3-dependent cell inflammatory responses	
Pancreatic cancer	⁃ SPHK1 expression was upregulated in pancreatic adenocarcinoma ductal lesions	Guillermet-Guibert et al. (2009), Aoki et al. (2016), Yu et al. (2021)
	⁃ SPHK1 expression was higher in metastatic pancreatic cancer tissues compared with normal pancreatic tissue and significant correlated with HAS2 expression	
	⁃ Knockdown of SPHK1 sensitized pancreatic cancer cells to gemcitabine-induced cell death	
	⁃ *SphK1* knockout mice exhibited lower tumor burden and fewer pancreatic cancer peritoneal carcinomatosis nodules 2 weeks after implantation	

AKT, protein kinase B; ALDH1, aldehyde dehydrogenase 1; Bax, Bcl-2-associated X; Bcl-2, B-cell lymphoma 2; Bcl-xl, B-cell lymphoma-extra-large; Bim, Bcl-2-like 11; FoxO3a, Forkhead box class O 3a; c-IAP, cellular inhibitor of apoptosis protein; ERK, extracellular signal regulated kinase; FSCN1, fascin; G3BP, Ras GTPase-activating protein-binding protein; HAS2, hyaluronan synthases 2; MAPK, mitogen-activated protein kinase; NF-κB, nuclear factor-κB; OCT4, octamer-binding transcription factor 4; p65 PAK1, p21-activated protein kinase 1; PI3K, phosphoinositide 3-kinase; S1PR, sphingosine-1-phosphate receptor; SOX2, SRY (sex determining region Y)-box 2; STAT, signal transducer and activator of transcription; TRAF1, tumor necrosis factor receptor associated factor.

In fact, compelling evidence has indicated that the oncogenic signaling cascades driven by SPHK1 is highly dependent on its activation and translocation to plasma membrane. In other words, translocation of the cytosolic SPHK1 to the plasma membrane is required for oncogenic activity as this enhances catalytic activity. This process can be stimulated through phosphorylation of SPHK1 on serine 225 (Ser225) by extracellular signal-regulated kinases 1 and 2 (ERK1/2). In a pioneering study, Pitson et al. (2005) confirmed that the oncogenic activity of SPHK1 is dependent on Ser225 phosphorylation-driven translocation (Pitson et al., 2005). They showed that Ser225 phosphorylation-deficient SPHK1 mutant cells failed to re-localize cytosolic SPHK1 to the plasma membrane and did not exhibit the oncogenic effects of SPHK1 overexpression, despite the intrinsic catalytic activity was retained in phosphorylation-deficient form of the enzyme (Pitson et al., 2005). Additionally, calcium and integrin binding protein 1 (CIB1) by exerting its function as Ca^2+^-myristoyl switch, is responsible for further facilitating plasma membrane localization of SPHK1, potentiating the membrane-associated enzymatic activity of SPHK1, as well as stimulating its oncogenic signaling activity (Jarman et al., 2010; Zhu et al., 2017). Although interactions with other molecules, for instance phosphatidic acid, filamin A, tumor necrosis factor (TNF) receptor-associated factor 2 (TRAF2), aminoacylase 1, and protein elongation factor 1A (eEF1A), have also been implicated in the regulation of SPHK1 activity or its localization at the plasma membrane (Xia et al., 2002; Maceyka et al., 2004; Leclercq et al., 2008), the significance of these interactions in SPHK1-driven oncogenic signaling remains to be validated.

## SPHK1 Signaling in Breast Cancer

The translocation of activated SPHK1 to plasma membrane leads to the generation of S1P, which subsequently acts as a bioactive sphingolipid to regulate cancer cell growth and metastasis. S1P can be secreted from cancer cells via specific transporters like protein spinster homolog 2 (SPNS2) and ABC transporters, of which the latter involve ABC sub-family A member 1 (ABCA1), ABC sub-family C member 1 (ABCC1), and ABC sub-family G member 2 (ABCG2) (Geffken and Spiegel, 2018). Once the intracellular S1P is released through the transporter, it usually binds to one of the G protein-coupled S1PRs (S1PR1 to S1PR5) to elicit oncogenic sphingolipid signaling in an autocrine or paracrine manner. Of note, it is uncommon to have all S1PRs to be expressed on the plasma membrane simultaneously, by which their expressions are subjected to the maturation stages of the cells and tissues (Strub et al., 2010). The engagement of S1P with different S1PRs will trigger context-dependent cell growth, migration, and invasion via distinctive signal transduction pathways. Interestingly, “criss-cross” pathway activations often occur when bindings of S1P to S1PRs stimulate the receptor tyrosine kinases (RTKs) involved in the cancer proliferation and angiogenesis, for instance vascular endothelial growth factor (VEGF), epidermal growth factor receptor (EGFR), and platelet-derived growth factor receptor (PDGFR), and at the same time the growth factors involved in these RTK activations can also enhance SPHK1 activity (Geffken and Spiegel, 2018).

In the context of breast cancer, it has been shown that high tumor expression of SPHK1 is associated with poorer disease outcomes in breast cancer patients across different subtypes (Alshaker et al., 2020). Studies have reported that SPHK1 expression is increasing in trend along with the advancement of breast tumor staging; while basal-like triple negative breast cancer (TNBC) tumors exhibit the highest levels of SPHK1 when compared to other subtypes. Notably, SPHK1 expression in cancerous cells is at least two-fold greater than adjacent normal tissues obtained from the same patients (French et al., 2003; Datta et al., 2014; Wang et al., 2018; Acharya et al., 2019).

**FIGURE 3:**
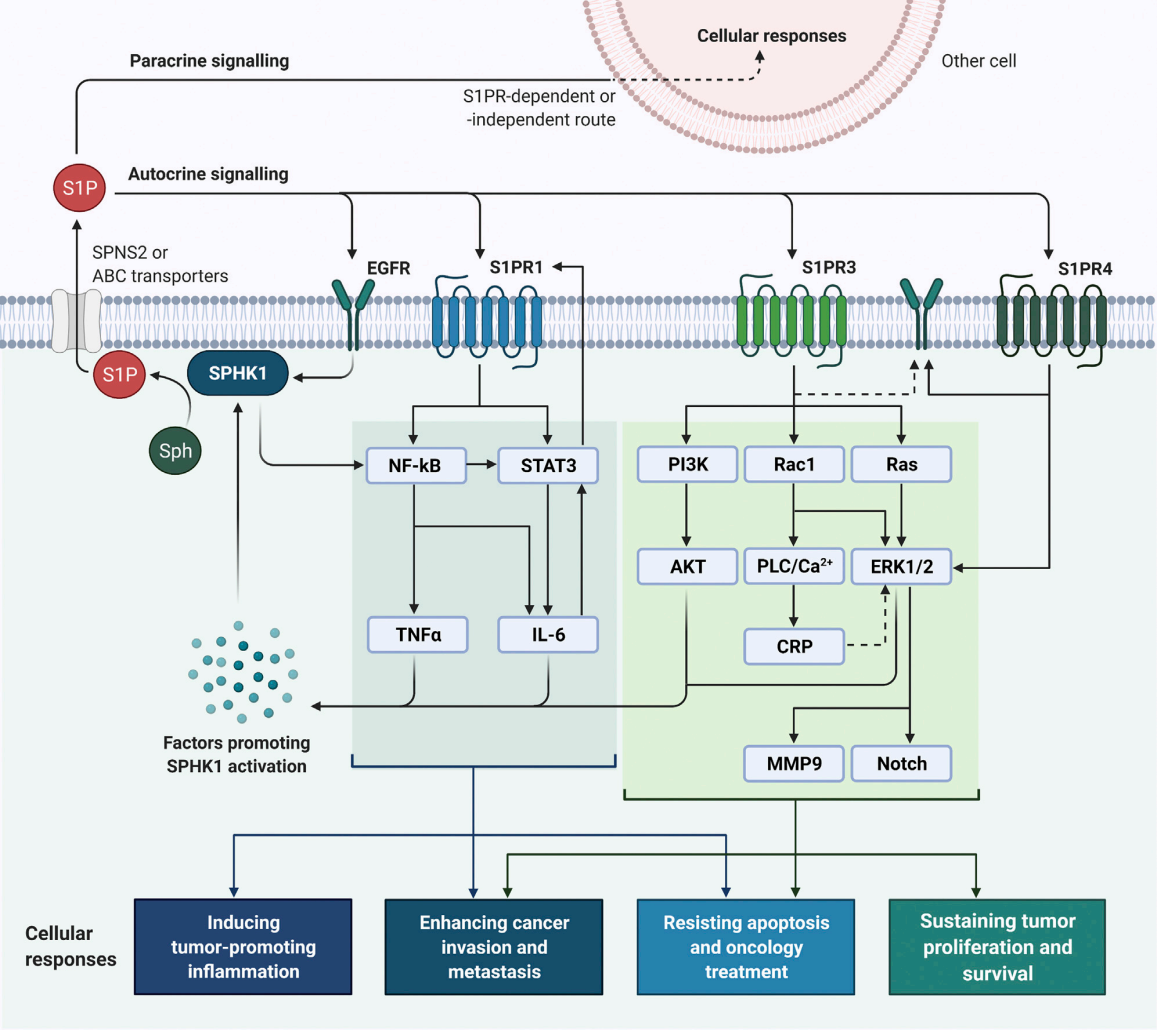
SPHK1/S1P signaling in breast cancer cells. SPHK1 activity can be activated upon stimulations by factors (e.g., estrogen, EGF, proinflammatory cytokines, calcium and protein kinase activators) and mechanisms (e.g., ERK1/2-induced phosphorylation, transactivation of EGFR, and NF-κB activation), leading to S1P formation (Geffken and Spiegel, 2018; Alshaker et al., 2020). Once the intracellular S1P is released via specific transporters, it can bind to S1PRs and EGFR to initiate oncogenic signaling in breast cancer through an autocrine or paracrine manner. Binding of S1P to S1PR1 can activate NF-κB and STAT3 signaling and induce production of pro-inflammatory cytokines involving IL-6 and TNFα; reciprocally, IL-6 potentiates STAT3 activity and STAT3 enhances the expression of S1PR1 STAT3 (Lee et al., 2010; Liang et al., 2013; Alshaker et al., 2014; Alshaker et al., 2015). Activation of SPHK1/S1P/S1PR3 axis is prominent in breast cancer and plays significant roles in breast tumorigenesis by activating PI3K/AKT, Rac1/PLC/Ca^2+^/CRP, Ras-dependent ERK1/2, MMP9 and Notch signaling pathways (Kim et al., 2011; Kim et al., 2014; Wang et al., 2018). As for S1PR4, it is found that SPHK1/S1P/S1PR4 axis regulates EGFR (specifically HER2) and ERK1/2 activation in breast cancer (Long et al., 2010b; Ohotski et al., 2012). ABC, ATP-binding cassette; CRP, C-reactive protein; EGF, epidermal growth factor; EGFR, EGF receptor; ERK1/2, extracellular signal-regulated kinases 1 and 2; IL-6, interleukin-6; MMP9, matrix metalloproteinase 9; NF-κB, nuclear factor-κB; PI3K, phosphoinositide 3-kinase; PLC, phospholipase C; S1P, sphingosine-1-phosphate; S1PR, sphingosine-1-phosphate receptor; Sph, sphingosine; SPHK1, sphingosine kinase 1; SPNS2, protein spinster homologue 2; STAT3, signal transducer and activator of transcription 3; TNFα, tumor necrosis factor α.

Also, clinical observations have shown that estrogen receptor (ER)-positive breast cancer patients whose tumors harboring high SPHK1 expression are generally less sensitive to tamoxifen treatment; whereas increased levels of SPHK1 are often detected in ER-negative breast tumors that failed chemotherapy as compared to those complete respondents, suggesting SPHK1 expression confers drug resistance in breast cancer (Long et al., 2010a; Watson et al., 2010; Datta et al., 2014). Consistent with clinical findings, studies have demonstrated that targeting SPHK1 using RNAi-mediated approaches or pharmacological SPHK1 inhibitors can reduce cell proliferation, induce apoptosis, synergize chemo- and endocrine sensitivity, impede invasion, and decrease metastasis in breast cancer (Antoon et al., 2011; Datta et al., 2014; Sukocheva and Wadham, 2014; Wang et al., 2018; Acharya et al., 2019). Importantly, it is evident that specific SPHK1 inhibition does not affect the growth and viability of non-cancerous breast epithelial cells (Antoon et al., 2011; Datta et al., 2014). Many have also attempted to investigate the involvement of S1PRs in the signaling cascades of SPHK1/S1P axis in human cancers, whereby substantial evidence has highlighted the interactions between SPHK1/S1P axis with S1PR1, S1PR3, and S1PR4 in breast cancer (Figure 3).

### SPHK1/S1PR1 Signaling in Breast Cancer

Ohotski et al. (2013) have revealed that high co-expression of SPHK1 and S1PR1 is correlated with poorer survival in patients with ER-positive breast cancer (Ohotski et al., 2013). In fact, S1PR1 has been found to persistently activate signal transducer and activator of transcription 3 (STAT3), of which STAT3 has been known to trigger various pathways that promote acquisition of cancer hallmarks (Lee et al., 2010; Liu et al., 2012). It is worth mentioning that STAT3 also promotes transcriptional activities of S1PR1 and the binding of S1P to S1PR1 can reciprocally activate STAT3 (Lee et al., 2010; Liang et al., 2013; Alshaker et al., 2014; Alshaker et al., 2015).

In breast cancer, incessant activation of STAT3 appears to be attributable to the upregulation of the pro-inflammatory cytokine IL-6 and S1PR1 (Lee et al., 2010; Alshaker et al., 2014; Alshaker et al., 2015). Alshaker and colleagues demonstrated that leptin as an adipokine was able to induce upregulation of SPHK1 and in turn SPHK1 could contribute to leptin-induced STAT3 activity in breast cancer via transactivation of IL-6/gp130 (Alshaker et al., 2014; Alshaker et al., 2015). Furthermore, studies have suggested that SPHK1/S1P/S1PR1 signaling pathway also aids to mediate treatment-induced inflammation and resistance to doxorubicin and tamoxifen in breast cancer, which is also accompanying with upregulation of NF-κB/IL-6/STAT3 (Katsuta et al., 2017).

Recently, Nagahashi et al. (2018) have reported a critical function of SPHK1/S1P/S1PR signaling in connecting obesity-induced chronic inflammation to breast oncogenesis and metastasis via activation of NF-κB and STAT3, along with production of IL-6 and TNFα (Nagahashi et al., 2018). Taken together, it is believed that SPHK1/S1P/S1PR1 axis by interacting with inflammatory cytokine amplification loops, could have prominent roles in the progression of inflammation-driven breast cancers.

### SPHK1/S1PR3 Signaling in Breast Cancer

S1PR3 is the most highly expressed S1PR in human breast cancer cells. Indeed, clinical observations have shown that elevated S1PR3 expression is closely associated with worse prognosis in patients with ER-positive breast cancer (Goetzl et al., 1999; Watson et al., 2010; Hirata et al., 2014; Wang et al., 2018). The S1PR3-driven oncogenic effects are mainly ascribed to the activation of ERK1/2 signaling pathways, which have been known as key regulatory mechanisms in the cell cycle progression, survival, and proliferation of breast cancer cells (Watson et al., 2010; Datta et al., 2014; Wang et al., 2016). SPHK1/S1P/S1PR3 axis has been shown to trigger the accumulation of phosphorylated ERK1/2 and actin into membrane ruffles/lamellipodia and inducing a refractory migratory phenotype in ER-positive breast cancer cells (Long et al., 2010a). Notably, knockdown of SPHK1 has been found to suppress S1PR3 expression and retard S1P/S1PR3-dependent ERK1/2 activation, inferring a functional regulation between SPHK1/S1P/S1PR3 axis and ERK1/2 signaling in ER-positive breast cancer (Long et al., 2010a). Though not specifying which S1PR, findings from Datta et al. (2014) have suggested that SPHK1/S1P/S1PR signaling pathway functions to stimulate and sustain the activation of ERK1/2 and AKT for the growth of TNBC cells (Datta et al., 2014).

In addition to ERK, SPHK1/S1P/S1PR3 axis has been implicated in potentiating the progression of breast cancer through upregulations of C-reactive protein (CRP) and MMP9. It has been demonstrated that coupling of S1P to S1PR3 can induce CRP expression via CCAAT/enhancer-binding protein β (C/EBPβ), activate Rac1/Nox-4/ROS/ERK pathways, and upregulate MMP9 activity, contributing to the aggressiveness and invasion of breast cancer (Kim et al., 2011; Kim et al., 2014).

On the other hand, Sukocheva and colleagues have revealed the involvement of SPHK1/S1P/S1PR3 axis in estrogen-induced EGFR transactivation in mitogenic stimulation of ER-positive breast cancer, by which S1P generated by the estrogen-induced SPHK1 binds to S1PR3 and activate EGFR in a Src/MMP-dependent manner (Sukocheva et al., 2006; Sukocheva and Wadham, 2014). Moreover, studies have indicated that SPHK1/S1P/S1PR3 signaling axis promotes tumorigenicity and metastasis of breast cancer by activating p38 mitogen-activated protein kinase (MAPK)/Notch signaling (Hirata et al., 2014; Wang et al., 2018).

### SPHK1/S1PR4 Signaling in Breast Cancer

As for S1PR4, a clinical study has shown that higher tumor expressions of SPHK1 and S1PR4 are associated with shorter disease-free survival and more advanced lymph node status in ER-negative breast cancer patients (Ohotski et al., 2012). Importantly, studies have demonstrated that SPHK1 and S1PR4 are functionally linked in mediating the survival of ER-negative breast cancer cells and SPHK1/S1P/S1PR4 axis interacts with HER2 to regulate ERK-1/2 pathways, implying a rationale of targeting S1PR4 in ER-negative HER2-positive breast cancer (Long et al., 2010b; Ohotski et al., 2012). Intriguingly, it has been shown that S1PR4 acts to prevent nuclear translocation of S1PR2 in ER-negative breast cancer, but this mechanism appears to be driven by SPHK2-derived S1P and some have suggested S1PR2 may counteract the oncogenic function of SPHK1 (Pyne et al., 2012; Ohotski et al., 2014). Nonetheless, the detailed mechanisms of how SPHKs and/or S1PR4 regulate S1PR2, and the significance of S1PR2 in breast oncogenesis, are yet to be elucidated and warrant more investigations.

### S1PR-Independent Signaling Pathways in Breast Cancer

Aside from S1PR-dependent signaling pathways, SPHK1/S1P axis also facilitates cancer cell signaling without involvement of S1PRs. In pathogenesis of ER-positive breast cancer, SPHK1/S1P axis and estrogen receptor signaling have been known to interact with one another, leading to enhanced tumor growth and therapy resistance (Sukocheva and Wadham, 2014). Early findings have revealed that overexpression of SPHK1 potentiates tumorigenesis of ER-positive breast cancer in estrogen and S1P-dependent manner, along with ERK1/2 activation (Nava et al., 2002). Also, studies have reported the ability of estrogen and EGF in upregulating SPHK1 activity in ER-positive breast cancer cells, as well as the capacity of estrogen and S1P in activating EGFR at plasma membrane (Sukocheva et al., 2003; Döll et al., 2005; Sarkar et al., 2005; Pinho et al., 2013). The switch from estrogen/ER-mediated tumorigenesis to SPHK1/S1P/EGFR-activated tumor growth has been regarded as important mechanism for acquiring endocrine resistance in ER-positive breast cancer (Sukocheva and Wadham, 2014; Maczis et al., 2016; Maczis et al., 2018). Moreover, S1P has been found to act with insulin-like growth factor binding protein 3 (IGFBP-3) to promote EGFR signaling for the progression of TNBC (Martin et al., 2014). On the other hand, some have proposed a link between SPHK1/S1P axis with protein kinase C (PKC) activity in TNBC, of which targeting SPHK1 in TNBC can suppress cell proliferation and survival by compromising SPHK1/S1P/PKC signaling pathway (Kotelevets et al., 2012). Moreover, Acharya et al. (2019) have reported that SPHK1 promotes TNBC metastasis by transcriptionally upregulating the expression of a metastasis-promoting gene, FSCN1 via activation of NF-κB (Acharya et al., 2019). Such activation of SPHK1/NF-κB/FSCN1 signaling pathway is probably attributable to SPHK1-generated S1P that has been established as a cofactor of TRAF2 to stimulate receptor-interacting serine/threonine-protein kinase 1 (RIPK1) and NF-κB activation (Alvarez et al., 2010).

In summary, the signaling network modulated by SPHK1/S1P axis is interwoven with various S1PR-dependent and S1PR-independent signaling pathways in breast cancer cells. Indeed, these mechanisms are further complicated by the “cross-activations” and overlapping regulators between the signaling cascades. It is believed that these interactions could be of dynamic along the cancer progression, whereby more studies are required for mechanistic clarifications in the context of breast cancer. Nevertheless, evidence to date has uniformly supported a rationale of targeting SPHK1/S1P axis in breast cancer which warrants further therapeutic development.

## SPHK1/S1P Signaling in Cancer Stem Cells

Despite extensive efforts have been put in mapping out the signaling network of sphingolipid rheostat in non-stem cancer cells, the specific roles of SPHK1/S1P axis in both normal and CSC biology are just started to emerge. To date, most of the understanding on the function of sphingolipids in human stem cells is derived from the attempts that evaluated their involvement in normal tissue homeostasis.

As for S1P, it has been shown to mediate proliferation and maintain the multipotency of different types of human stem cells, including human embryonic stem cells, neural progenitor cells and bone marrow-derived stem cells, via mechanisms involved platelet-derived growth factor (PDGF) and ERK signaling (Pébay et al., 2005; Inniss and Moore, 2006; Wong et al., 2007; Avery et al., 2008; Rodgers et al., 2009; He et al., 2010). In addition to ERK activation, treatment of S1P in neural progenitor cells obtained from rat embryos has been found to induce telomerase activity, implying S1P might possess parallel role in maintaining stem cells across different species (Harada et al., 2004). Moreover, it has been demonstrated that S1P promotes proliferation of mouse embryonic stem cells by eliciting transactivation of fetal liver kinase-1 (FLK-1) through stimulation of S1PR1/S1PR3-dependent β-arrestin/c-Src pathways and ERK/c-Jun N-terminal kinase (JNK) activation (Ryu et al., 2014). Interestingly, studies have revealed that S1PR2 functions to inhibit clonogenicity, migration, and proliferation of mesenchymal stem cells (MSCs) by suppressing ERK phosphorylation; whereas inhibition of S1PR2 triggers PDGF-induced migratory response of mouse embryonic fibroblasts and knockdown of S1PR1 can abrogate the migration induced by knockout of S1PR2 in mouse embryonic fibroblasts (Goparaju et al., 2005; Price et al., 2015). Although it has been reported that many stem cell types are expressing S1PRs, their functions in these cells remain largely unknown at present and warrant further investigations (Pébay et al., 2007). Nonetheless, based on current evidence available, it is generally believed that S1PR1 and S1PR3 play roles in promoting self-renewal and proliferation of normal stem cells.

Compared to the regulatory roles of sphingolipid rheostat in normal stem cell biology, little is known about its functions in CSCs. Since CSCs exhibit stem-like traits akin to normal stem cells, it is not uncommon that those pathways which have been well-characterized in normal stem cell maintenance are hijacked during oncogenesis, notably Wnt/β-Catenin, Notch, Hedgehog, and Hippo pathways. Whilst the signaling axis of SPHK1/S1P has been implicated in the maintenance of normal stem cells, several pieces of evidence have also indicated the involvement of SPHK1/S1P axis in CSC-driven tumorigenesis. In the context of breast cancer, Nava et al. (2002) first suggested a role of SPHK1 in ER-positive breast tumorigenesis when they observed that ER-positive breast cancer cells with SPHK1 overexpression were highly tumorigenic with enhanced capability to induce larger breast tumors in mice (Nava et al., 2002). This finding could be indicative of the association between SPHK expression and the emergence of highly tumorigenic cells that capable of initiating and sustaining tumor growth *in vivo*, which are key characteristics of breast CSCs. Multiple clinical observations have subsequently reported the contribution of high SPHK1 expression to therapy resistance, disease recurrence and metastasis in breast cancer patients (Long et al., 2010a; Watson et al., 2010; Ohotski et al., 2012; Do et al., 2017; Acharya et al., 2019), all of which are similar to the clinical implications of breast CSCs. As none of these studies further stratified these SPHK1-expressing breast tumors based on CSC markers, the contribution of SPHK1 towards breast CSCs could be largely overlooked. It is conceivable that SPHK1 may exert roles in the enrichment and/or maintenance of breast CSC subpopulation, conferring the breast tumor with enhanced tumorigenicity and resistance towards oncology treatment, and thus leading to relapse and metastasis.

On the other hand, some pre-clinical studies of breast cancer have described the interactions between SPHK1 and numerous signaling targets which have been known as pivotal players in the regulation of breast CSCs, such as Notch, NF-κB, STAT3, EGFR, and MMPs (Lee et al., 2010; Kim et al., 2011; Marotta et al., 2011; Hirata et al., 2014; Nagahashi et al., 2018), suggesting that SPHK1 is as a potential functional target in breast CSCs. While most of them are yet to be validated using CSC models, this notion has begun to gain support from recent findings that have indicated a role of SPHK1/S1P/S1PR3 axis in regulating ER-positive breast CSCs via Notch signaling. In this regard, Hirata et al. (2014) have pioneered to demonstrate that SPHK1/S1P axis acts to promote CSC formation via activation of S1PR3/Notch signaling axis in ER-positive breast cancer, and most importantly, they have found that the tumorigenicity of ALDH^+^ breast CSCs can be enhanced by increased expression of SPHK1 in a S1PR3-dependent manner (Hirata et al., 2014). Likewise, a separate study by Wang et al. (2016) have also revealed a stimulatory function of S1P on breast CSCs in ER-positive breast cancer, whereby they showed that phthalates as environmental carcinogens, could augment CSC-driven metastasis in ER-positive breast cancer model by activating SPHK1/S1P/S1PR3 signaling (Wang et al., 2016). More recently, Sukocheva et al. (2021) reported the increased expression of S1PR3 in ER-positive breast CSCs and further suggested the involvement of TNFα signaling in regulating the intracellular trafficking of SPHK1 and S1PR3 in these CSCs (Sukocheva et al., 2021). Moreover, another study showed that ectopic expression of SPHK1 contributed to the maintenance of mammary stem cell-like characteristics in ER-positive breast cancer (Chen and Liu, 2020).

While earlier reports have inferred the functions of SPHK1/S1P axis in promoting the growth, tumorigenicity and metastasis of ER-positive breast CSCs, recent evidence has further highlighted that the activation of SPHK1/S1P axis is greater in CSCs than non-stem cancer cells across different subtypes of breast cancers (Hii et al., 2020). For TNBC, which lack of expression, it is reported that SPHK1 acts to promote TNBC CSC survival by attenuating interferon/STAT1 signaling (Hii et al., 2020). Notably, selective SPHK1 inhibition, but not SPHK2, has been shown to synergize doxorubicin sensitivity in breast CSCs derived from TNBC (Hii et al., 2020). Collectively, these findings indicate that SPHK1 plays a functional role in regulating breast CSCs derived from different subtypes, regardless of ER expression. Nevertheless, it is anticipated that SPHK1 would act via different signalling pathways to regulate the survival of CSCs derived from different subtypes of breast cancer. The dependency of breast CSCs on SPHK1 underscores the great promise of targeting SPHK1 in the treatment of refractory breast cancer.

## Current Therapeutics Targeting SPHK1

**FIGURE 4:**
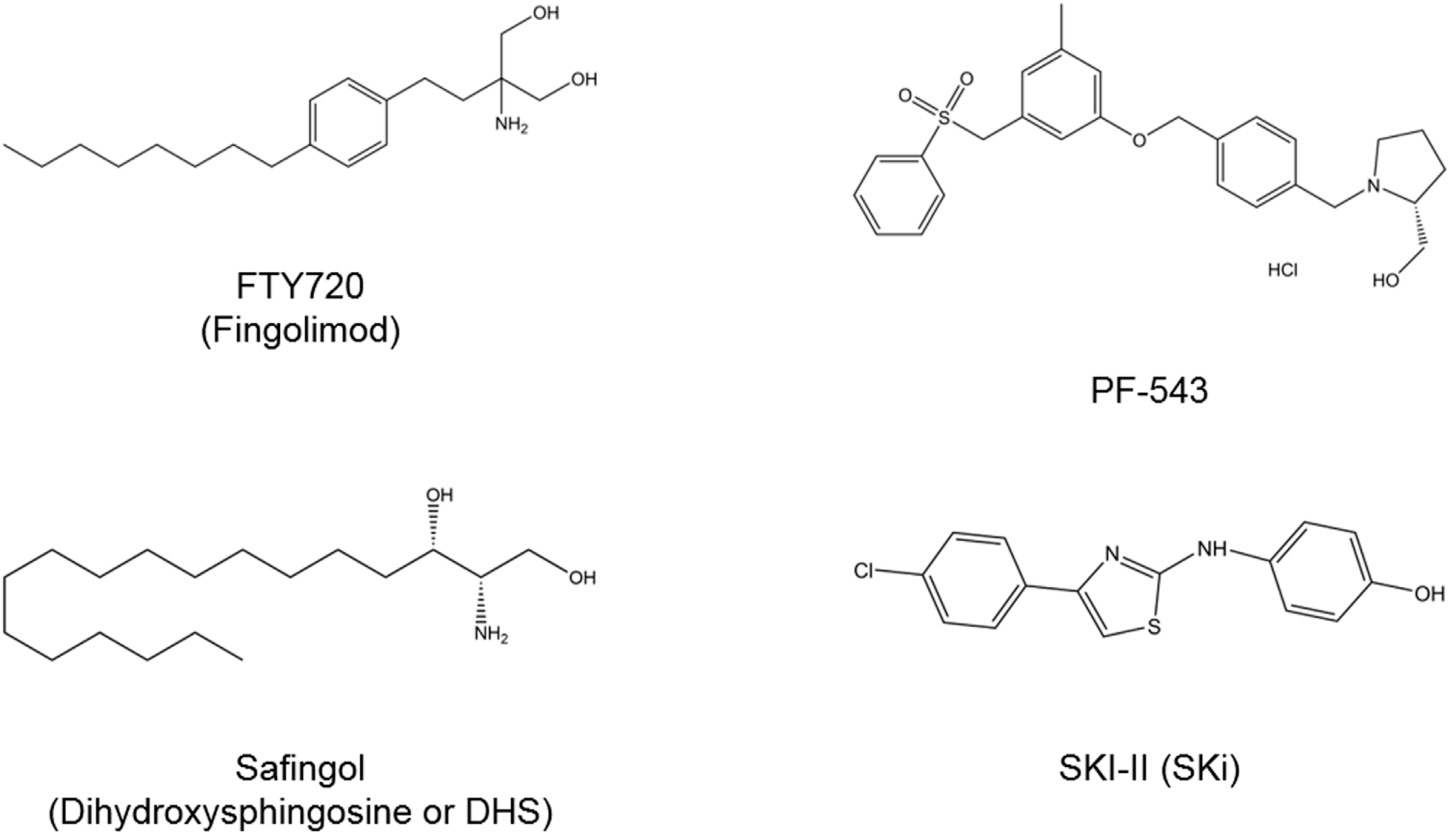
Chemical structures of SPHK inhibitors. FTY720 acts to inhibit SPHK1 and modulate S1PR. Safingol is a non-selective SPHK and PKC inhibitor. SKI-II is a dual SPHK1/SPHK2 inhibitor and PF-543 is a selective SPHK1 inhibitor. PKC, protein kinase C; S1PR, sphingosine-1-phosphate receptor; SPHK, sphingosine kinase.

As compelling evidence has implicated of SPHK/S1P rheostat in oncogenesis, there is growing interest to develop and exploit the therapeutics targeting this signaling axis as anti-cancer therapies. Numerous bioactive small molecules have been developed to modulate SPHK/S1P/S1PR signaling, including SPHK inhibitors, anti-S1P antibody, and S1PR modulators (Figure 4) (Alshaker et al., 2020). Of all SPHK1 inhibitors developed to date, only safingol has entered oncology-related clinical trials (Table 2).

**TABLE 2:** Current clinical status of therapeutics targeting SPHK1.

Agent	Mechanism of action	Highest stage of development	Notes	References
FTY720	SPHK1 inhibitor and S1PR modulator	Phase IV	⁃ FDA-approved for relapsing multiple sclerosis	Strader et al. (2011)
			⁃ No clinical trials available to evaluate it as anti-cancer drug	
Safingol	SPHK1 and SPHK2 inhibitor	Phase I	⁃ Also well-known as a PKC inhibitor	Dickson et al. (2011)
			⁃ Its phase I study in patients with advanced stages of solid malignancies reported that it can be given as combination therapy with cisplatin with reversible and manageable dose-dependent hepatotoxicity (ClinicalTrials.gov Identifier: NCT00084812)	
			⁃ Another phase I clinical trial is underway to evaluate safingol as combination therapy with fenretinide in patients with relapsed malignancies (ClinicalTrials.gov Identifier: NCT01553071)	
SKI-II	SPHK1 and SPHK2 inhibitor	Pre-clinical	⁃ Non-selective inhibitor of SPHK1 (Ki = 16 μM) and SPHK2 (Ki = 7.9 μM)	French et al. (2003), Quinn and Wang (2008)
			⁃ Failed to advance into clinical trials due to poor bioavailability	
PF-543	SPHK1 inhibitor	Pre-clinical	⁃ Most potent selective SPHK1 inhibitor with Ki = 3.6 nM with >100-fold selective towards SPHK1 over the SPHK2	Schnute et al. (2012), Lynch (2012)
			⁃ Serves as a useful agent for studying the specific roles of targeting SPHK1/S1P axis in pre-clinical disease models	

FDA, food and drug administration; mAb, monoclonal antibody; PKC, protein kinase C; SPHK, sphingosine kinase; S1P, sphingosine-1-phosphate; S1PR, sphingosine-1-phosphate receptor; VEGF, vascular endothelial growth factor.

### FTY720

FTY720 (also known as fingolimod) is a sphingosine analog that is known to inhibit SPHK1 and modulate S1PR (Tonelli et al., 2010; Haddadi et al., 2017). It is an orally available immunomodulatory drug that has received FDA approval for the treatment of relapsing multiple sclerosis (Strader et al., 2011). While being actively involved in clinical trials for expanding its indications in autoimmune and neuro-degenerative disorders, FTY720 also exhibits promising anti-cancer properties in various *in vitro* and *in vivo* cancer models, implying a potential of repurposing FTY720 for cancer treatment (Zhang et al., 2013; White et al., 2016).

In the context of breast cancer, pre-clinical studies have demonstrated the efficacy of FTY720 in suppressing the development and progression of breast cancer when given as monotherapy or as adjuvant therapy (Azuma et al., 2002; Tonelli et al., 2010; Lim et al., 2011; Hait et al., 2015; Rincón et al., 2015). In addition, FTY720 has been reported as chemo-sensitizer in non-stem cancer cells and CSCs (Rincón et al., 2015; White et al., 2016). It has been found that FTY720 can potentiate the chemotherapeutic effects of doxorubicin in breast cancer xenograft models, especially those with acquired resistance to doxorubicin (Rincón et al., 2015). Another study has revealed that FTY720 can enhance doxorubicin sensitivity in breast CSCs of TNBC (Hii et al., 2020). FTY720, by synergizing with TNF-related apoptosis inducer ligand (TRAIL), has also been shown to reduce tumor burden and induce tumor-specific apoptosis in xenograft breast cancer models without affecting normal cells (Woo et al., 2015).

In addition to its function against sphingolipid signaling, some have reported that part of anti-cancer effects of FTY720 can be attributed to its ability to induce re-activation of protein phosphatase 2A (PP2A), which is a tumor suppressor that is commonly repressed in breast cancer (Perrotti and Neviani, 2013; Saddoughi et al., 2013). Although currently there is no active clinical trial being designed to directly evaluate FTY720 as anti-cancer drug, it is anticipated that an ongoing pharmacovigilance study that aims to assess whether there is any association between the use of FTY720 and incidence of breast cancer development in patients with multiple sclerosis (ClinicalTrials.gov Identifier: NCT04237337), as well as a phase I trial investigating the clinical use of FTY720 as preventive measure for paclitaxel-associated neuropathy in patients with breast cancer (ClinicalTrials.gov Identifier: NCT03941743), will offer valuable insights on the roles of FTY720 in oncology settings in the near future.

### Safingol

The SPHK inhibitors that were developed in the early stages are simple analogues of sphingosine which are generally low-potency and not selective, of which safingol (also known as dihydroxysphingosine or DHS) is one of them (Santos and Lynch, 2015). Safingol is a sphingosine-competitive SPHK1 inhibitor (Olivera et al., 1998). Pre-clinical studies have demonstrated that the anti-tumor activities of safingol and its prominent efficacy in synergizing the killing effects of chemotherapeutic agents such as cisplatin, doxorubicin and irinotecan (Kedderis et al., 1995; Schwartz et al., 1995; Schwartz et al., 1997; Coward et al., 2009; Ling et al., 2009; Ling et al., 2011). However, it is important to note that the anti-cancer effects induced by safingol can also be attributable to its role as PKC inhibitor (Kedderis et al., 1995; Schwartz et al., 1995; Schwartz et al., 1997; Coward et al., 2009; Ling et al., 2009). Nevertheless, safingol is the first putative SPHK inhibitor that entered clinical trial as oncology therapeutic agent and it has completed its phase I trial as a combination therapy with cisplatin in patients with advanced stages of solid malignancies (ClinicalTrials.gov Identifier: NCT00084812) (Dickson et al., 2011). This trial has reported that it is safe to combine safingol with cisplatin and this combinatorial approach is able to achieve target inhibition with reduction in plasma S1P levels in a dose-dependent manner (Dickson et al., 2011). Though dose-dependent hepatotoxicity was observed in the trial, such hepatic toxicity events were reversible and manageable (Dickson et al., 2011). At present, safingol is being tested in another phase I clinical trial as combination therapy with fenretinide in patients experiencing cancer relapse (ClinicalTrials.gov Identifier: NCT01553071).

### SKI-II

Early screenings by French and colleagues have identified four SPHK inhibitors, namely SKI-I, SKI-II, SKI-III, and SKI-IV, of which SKI-II (also known as SKi) is the most studied compound than the other three (French et al., 2003; French et al., 2010). SKI-II is a sphingosine-competitive, non-selective inhibitor of SPHK1 (Ki = 16 μM) and SPHK2 (Ki = 7.9 μM) (French et al., 2003). It has been documented to suppress *in vitro* cell proliferation in different types of cancers, including breast cancer, pancreatic cancer, bladder cancer, and prostate cancer (French et al., 2010; Loveridge et al., 2010; Haddadi et al., 2017). Some have linked such anti-cancer effects to its abilities in inducing proteasomal and lysosomal degradation of SPHK1 (Loveridge et al., 2010; Ren et al., 2010). Besides, SKI-II was found to target dihydroceramide desaturase (Degs) in the *de novo* synthesis of ceramide, regulating the levels of dihydroceramide and ceramide as well as the downstream S1P (Cingolani et al., 2014). However, its mechanism of actions have been further complicated when recent studies reported that SKI-II also acts to promote the polyubiquitination of Degs, in which such polyubiquitinated forms of Degs are known to activate pro-survival pathways, including p38 MAPK, JNK, and X-box protein 1s (XBP-1s) (Alsanafi et al., 2018; Alsanafi et al., 2020). Although the poor bioavailability of SKI-II has halted its further clinical investigations, it is still actively being used in pre-clinical studies as a pharmacological modality for interrogating the biological roles of dual SPHK1/SPHK2 inhibition (Quinn and Wang, 2008). Moreover, its co-crystal structure with SPHK1 plays important role in guiding the structural based drug design and development for SPHK inhibitors (Santos and Lynch, 2015).

### PF-543

Among the inhibitors targeting SPHK1 available to date, PF-543 is the most potent selective SPHK1 inhibitor with Ki at 3.6 nM (Schnute et al., 2012). It is known to act as a reversible sphingosine-competitive inhibitor which possesses more than 100-fold selectivity towards SPHK1 than SPHK2 (Schnute et al., 2012). Despite having nanomolar potency against SPHK1, it did not show promising anti-cancer effects in early study by Schnute et al. (2012) who demonstrated that PF-543 had insignificant effect on the cancer cell growth, when they tested it in cancer cell lines at single concentration of 1 µM (Schnute et al., 2012). Recent studies have demonstrated that PF-543 exerts anti-cancer activities in models of colorectal cancer, head and neck squamous cell carcinoma (HNSCC), and metastatic breast cancer when given at higher concentrations (Ju et al., 2016; Hamada et al., 2017; Maiti et al., 2017). PF-543 has been found to induce apoptosis, necrosis, and autophagy in HNSCC cell lines at treatment dose of 25 µM (Hamada et al., 2017). According to Maiti et al. (2017), treatment of PF-543 (up to 10 µM) attenuated EGF-mediated cell growth, survival and migration in metastatic breast cancer cells via inhibition of AKT, ERK, and p38 MAPK pathways (Maiti et al., 2017). When treated across colorectal cancer cell panel at 2.5 or 10 µM, PF-543 significantly inhibited cell proliferation and induced necrotic cell death (Ju et al., 2016). A subsequent *in vivo* study revealed that PF-543 intravenous injection remarkably suppressed tumor growth and improved the mice survival without any signs of other apparent toxicities in the animals (Ju et al., 2016). In addition, PF-543 has been shown to act synergistically with doxorubicin to kill breast CSCs (Hii et al., 2020). At present, PF-543 has yet to be advanced into clinical testing, efforts are ongoing to modify and synthesize PF-543 derivatives for better therapeutic efficiency in oncology models. Nevertheless, PF-543 has been regarded as a useful drug for studying the specific roles of targeting SPHK1/S1P axis in pre-clinical disease models (Lynch, 2012).

## Conclusions and Future Perspectives

Overall, the potential roles of sphingolipid rheostat in breast CSC biology are relatively underappreciated, as compared to non-stem breast cancer cells. Despite some eagerly highlight the oncogenic roles of SPHK1/S1P axis in breast CSCs based on existing mechanisms established from non-stem cancer cells, specific investigations in the context of breast CSCs, are generally lacking nowadays. As such, it is necessary to design and conduct proper studies for functional evaluation, mechanistic exploration, and validation on SPHK1/S1P signaling pathways in suitable models of breast CSCs. Further clinical studies should have careful patient stratification for more meaningful clinical correlation of SPHK1/S1P axis with CSC expression. Besides, with the chemotactic functions of S1P in the immunomodulation and microenvironmental regulation, it is also tempting to speculate that SPHK1/S1P axis may aid to regulate the anti-tumor functions of various immune cells, assist immunosurveillance of breast CSCs, and create a conducive tumor microenvironment (TME) for CSC propagation and maintenance. In addition, inhibition of interferon/STAT1 signaling have been shown to regulate a multigenic program in breast cancer cell autonomous function and resistance against immune checkpoint blockade (Dunn et al., 2006; Khodarev et al., 2012; Benci et al., 2016; Legrier et al., 2016; Ahn et al., 2017; Doherty et al., 2017; Budhwani et al., 2018; Castiello et al., 2018). Taken together with the reported role of SPHK1 in mediating breast CSC survival through suppression of interferon/STAT1 signaling (Hii et al., 2020), these findings suggest that SPHK1 might also contribute to CSC resistance against immune checkpoint blockade, though this warrants further validation. In light of emerging rationales of targeting TME and immunology of breast CSCs, future works should consider assessing whether SPHK1/S1P axis plays significant roles in mediating CSC niche and anti-tumor immune response of breast CSCs. To this end, deeper understanding on how SPHK1/S1P-mediated signaling cascades work as a whole in the regulation of breast CSCs will assist the development of synergistic treatment modalities for effective eradication of CSCs.

Given the known functional roles of SPHK/S1P rheostat in human breast cancers, it is undeniable that targeting SPHK/S1P signaling axis represents another novel and innovative avenue in cancer treatment that warrants more research attention. While the majority of the SPHK inhibitors, particularly selective SPHK1 inhibitor, have yet to advance into oncology clinical trials, more efforts are indeed required to develop and optimize the therapeutics that act against SPHK/S1P axis for anti-cancer applications. In view of the complexity of sphingolipid metabolism and context-dependent functions, it is imperative to continue elucidating the specific roles of the enzymes and receptors involved in sphingolipid signaling in both non-stem cancer cells and CSCs from different cancer cell types. A better understanding of sphingolipid signaling, and its structure-function relationships will be fundamental to enabling the improvement of drug design for effective clinical translation.

## References

[B1] Acharya S. Yao J. Li P. Zhang C. Lowery F. J. Zhang Q. (2019). Sphingosine Kinase 1 Signaling Promotes Metastasis of Triple-Negative Breast Cancer. Cancer Res. 79 (16), 4211–4226. 10.1158/0008-5472.CAN-18-3803 31239273 PMC6697620

[B2] Ahn R. Sabourin V. Bolt A. M. Hébert S. Totten S. De Jay N. (2017). The Shc1 Adaptor Simultaneously Balances Stat1 and Stat3 Activity to Promote Breast Cancer Immune Suppression. Nat. Commun. 8, 14638. 10.1038/ncomms14638 28276425 PMC5347092

[B3] Alemany R. van Koppen C. J. Danneberg K. ter Braak M. Meyer zu Heringdorf D. (2007). Regulation and Functional Roles of Sphingosine Kinases. Naunyn-schmied Arch. Pharmacol. 374 (5), 413–428. 10.1007/s00210-007-0132-3 17242884

[B4] Alsanafi M. Kelly S. L. Jubair K. McNaughton M. Tate R. J. Merrill A. H. (2018). Native and Polyubiquitinated Forms of Dihydroceramide Desaturase Are Differentially Linked to Human Embryonic Kidney Cell Survival. Mol. Cel Biol 38 (23). 10.1128/MCB.00222-18 PMC6234290 30224516

[B5] Alsanafi M. Kelly S. L. McNaughton M. Merrill A. H. Jr Pyne N. J. Pyne S. (2020). The Regulation of P53, P38 MAPK, JNK and XBP-1s by Sphingosine Kinases in Human Embryonic Kidney Cells. Biochim. Biophys. Acta (Bba) - Mol. Cel Biol. Lipids 1865 (4), 158631. 10.1016/j.bbalip.2020.158631 31954175

[B6] Alshaker H. Krell J. Frampton A. E. Waxman J. Blyuss O. Zaikin A. (2014). Leptin Induces Upregulation of Sphingosine Kinase 1 in Oestrogen Receptor-Negative Breast Cancer via Src Family Kinase-Mediated, Janus Kinase 2-independent Pathway. Breast Cancer Res. 16 (5), 426. 10.1186/s13058-014-0426-6 25482303 PMC4303110

[B7] Alshaker H. Thrower H. Pchejetski D. (2020). Sphingosine Kinase 1 in Breast Cancer-A New Molecular Marker and a Therapy Target. Front. Oncol. 10, 289. 10.3389/fonc.2020.00289 32266132 PMC7098968

[B8] Alshaker H. Wang Q. Brewer D. Pchejetski D. (2019). Transcriptome-Wide Effects of Sphingosine Kinases Knockdown in Metastatic Prostate and Breast Cancer Cells: Implications for Therapeutic Targeting. Front. Pharmacol. 10, 303. 10.3389/fphar.2019.00303 30971929 PMC6445839

[B9] Alshaker H. Wang Q. Frampton A. E. Krell J. Waxman J. Winkler M. (2015). Sphingosine Kinase 1 Contributes to Leptin-Induced STAT3 Phosphorylation through IL-6/gp130 Transactivation in Oestrogen Receptor-Negative Breast Cancer. Breast Cancer Res. Treat. 149 (1), 59–67. 10.1007/s10549-014-3228-8 25481644

[B10] Alvarez S. E. Harikumar K. B. Hait N. C. Allegood J. Strub G. M. Kim E. Y. (2010). Sphingosine-1-phosphate Is a Missing Cofactor for the E3 Ubiquitin Ligase TRAF2. Nature 465 (7301), 1084–1088. 10.1038/nature09128 20577214 PMC2946785

[B11] Antoon J. W. Meacham W. D. Bratton M. R. Slaughter E. M. Rhodes L. V. Ashe H. B. (2011). Pharmacological Inhibition of Sphingosine Kinase Isoforms Alters Estrogen Receptor Signaling in Human Breast Cancer. J. Mol. Endocrinol. 46 (3), 205–216. 10.1530/jme-10-0116 21321095 PMC4007162

[B12] Aoki H. Aoki M. Katsuta E. Ramanathan R. Idowu M. O. Spiegel S. (2016). Host Sphingosine Kinase 1 Worsens Pancreatic Cancer Peritoneal Carcinomatosis. J. Surg. Res. 205 (2), 510–517. 10.1016/j.jss.2016.05.034 27664902 PMC5656061

[B13] Avery K. Avery S. Shepherd J. Heath P. R. Moore H. (2008). Sphingosine-1-phosphate Mediates Transcriptional Regulation of Key Targets Associated with Survival, Proliferation, and Pluripotency in Human Embryonic Stem Cells. Stem Cell Dev. 17 (6), 1195–1206. 10.1089/scd.2008.0063 18393631

[B14] Azuma H. Takahara S. Ichimaru N. Wang J. D. Itoh Y. Otsuki Y. (2002). Marked Prevention of Tumor Growth and Metastasis by a Novel Immunosuppressive Agent, FTY720, in Mouse Breast Cancer Models. Cancer Res. 62 (5), 1410–1419. 11888913

[B15] Benci J. L. Xu B. Qiu Y. Wu T. J. Dada H. Twyman-Saint Victor C. (2016). Tumor Interferon Signaling Regulates a Multigenic Resistance Program to Immune Checkpoint Blockade. Cell 167 (6), 1540–1554.e12. 10.1016/j.cell.2016.11.022 27912061 PMC5385895

[B16] Budhwani M. Mazzieri R. Dolcetti R. (2018). Plasticity of Type I Interferon-Mediated Responses in Cancer Therapy: From Anti-tumor Immunity to Resistance. Front. Oncol. 8, 322. 10.3389/fonc.2018.00322 30186768 PMC6110817

[B17] Castiello L. Sestili P. Schiavoni G. Dattilo R. Monque D. M. Ciaffoni F. (2018). Disruption of IFN-I Signaling Promotes HER2/Neu Tumor Progression and Breast Cancer Stem Cells. Cancer Immunol. Res. 6 (6), 658–670. 10.1158/2326-6066.CIR-17-0675 29622580

[B18] Chen Z. Liu B. (2020). Sphk1 Participates in Malignant Progression of Breast Cancer by Regulating Epithelial-Mesenchymal Transition and Stem Cell Characteristics. Tissue and Cell 65, 101380. 10.1016/j.tice.2020.101380 32746988

[B19] Chipuk J. E. McStay G. P. Bharti A. Kuwana T. Clarke C. J. Siskind L. J. (2012). Sphingolipid Metabolism Cooperates with BAK and BAX to Promote the Mitochondrial Pathway of Apoptosis. Cell 148 (5), 988–1000. 10.1016/j.cell.2012.01.038 22385963 PMC3506012

[B20] Cingolani F. Casasampere M. Sanllehí P. Casas J. Bujons J. Fabrias G. (2014). Inhibition of Dihydroceramide Desaturase Activity by the Sphingosine Kinase Inhibitor SKI II. J. Lipid Res. 55 (8), 1711–1720. 10.1194/jlr.m049759 24875537 PMC4109765

[B21] Coward J. Ambrosini G. Musi E. Truman J.-P. Haimovitz-Friedman A. Allegood J. C. (2009). Safingol (L-Threo-Sphinganine) Induces Autophagy in Solid Tumor Cells through Inhibition of PKC and the PI3-Kinase Pathway. Autophagy 5 (2), 184–193. 10.4161/auto.5.2.7361 19098447

[B22] Datta A. Loo S. Y. Huang B. Wong L. Tan S. S. L. Tan T. Z. (2014). SPHK1 Regulates Proliferation and Survival Responses in Triple-Negative Breast Cancer. Oncotarget 5 (15), 5920–5933. 10.18632/oncotarget.1874 25153718 PMC4171602

[B23] Dickson M. A. Carvajal R. D. Merrill A. H. Gonen M. Cane L. M. Schwartz G. K. (2011). A Phase I Clinical Trial of Safingol in Combination with Cisplatin in Advanced Solid Tumors. Clin. Cancer Res. 17 (8), 2484–2492. 10.1158/1078-0432.Ccr-10-2323 21257722 PMC3078945

[B24] Do S. I. Kim H. S. Kim K. Lee H. Do I. G. Kim D. H. (2017). Predictive and Prognostic Value of Sphingosine Kinase 1 Expression in Patients with Invasive Ductal Carcinoma of the Breast. Am. J. Transl Res. 9 (12), 5684–5695. 29312521 PMC5752919

[B25] Doherty M. R. Cheon H. Junk D. J. Vinayak S. Varadan V. Telli M. L. (2017). Interferon-beta Represses Cancer Stem Cell Properties in Triple-Negative Breast Cancer. Proc. Natl. Acad. Sci. USA 114 (52), 13792–13797. 10.1073/pnas.1713728114 29229854 PMC5748193

[B26] Döll F. Pfeilschifter J. Huwiler A. (2005). The Epidermal Growth Factor Stimulates Sphingosine Kinase-1 Expression and Activity in the Human Mammary Carcinoma Cell Line MCF7. Biochim. Biophys. Acta 1738 (1-3), 72–81. 10.1016/j.bbalip.2005.12.001 16414307

[B27] Dunn G. P. Koebel C. M. Schreiber R. D. (2006). Interferons, Immunity and Cancer Immunoediting. Nat. Rev. Immunol. 6 (11), 836–848. 10.1038/nri1961 17063185

[B28] French K. J. Schrecengost R. S. Lee B. D. Zhuang Y. Smith S. N. Eberly J. L. (2003). Discovery and Evaluation of Inhibitors of Human Sphingosine Kinase. Cancer Res. 63 (18), 5962–5969. 14522923

[B29] French K. J. Zhuang Y. Maines L. W. Gao P. Wang W. Beljanski V. (2010). Pharmacology and Antitumor Activity of ABC294640, a Selective Inhibitor of Sphingosine Kinase-2. J. Pharmacol. Exp. Ther. 333 (1), 129–139. 10.1124/jpet.109.163444 20061445 PMC2846016

[B30] Fuereder T. Hoeflmayer D. Jaeger-Lansky A. Rasin-Streden D. Strommer S. Fisker N. (2011). Sphingosine Kinase 1 Is a Relevant Molecular Target in Gastric Cancer. Anticancer Drugs 22 (3), 245–252. 10.1097/cad.0b013e328340bd95 21360847

[B31] Fukuda Y. Kihara A. Igarashi Y. (2003). Distribution of Sphingosine Kinase Activity in Mouse Tissues: Contribution of SPHK1. Biochem. Biophysical Res. Commun. 309 (1), 155–160. 10.1016/s0006-291x(03)01551-1 12943676

[B32] Geffken K. Spiegel S. (2018). Sphingosine Kinase 1 in Breast Cancer. Adv. Biol. Regul. 67, 59–65. 10.1016/j.jbior.2017.10.005 29055687 PMC5807162

[B33] Goetzl E. J. Dolezalova H. Kong Y. Zeng L. (1999). Dual Mechanisms for Lysophospholipid Induction of Proliferation of Human Breast Carcinoma Cells. Cancer Res. 59 (18), 4732–4737. 10493533

[B34] Goparaju S. K. Jolly P. S. Watterson K. R. Bektas M. Alvarez S. Sarkar S. (2005). The S1P 2 Receptor Negatively Regulates Platelet-Derived Growth Factor-Induced Motility and Proliferation. Mol. Cel Biol. 25 (10), 4237–4249. 10.1128/mcb.25.10.4237-4249.2005 PMC1087716 15870293

[B35] Guillermet-Guibert J. Davenne L. Pchejetski D. Saint-Laurent N. Brizuela L. Guilbeau-Frugier C. (2009). Targeting the Sphingolipid Metabolism to Defeat Pancreatic Cancer Cell Resistance to the Chemotherapeutic Gemcitabine Drug. Mol. Cancer Ther. 8 (4), 809–820. 10.1158/1535-7163.mct-08-1096 19372554

[B36] Haddadi N. Lin Y. Simpson A. Nassif N. McGowan E. (2017). “Dicing and Splicing” Sphingosine Kinase and Relevance to Cancer. Ijms 18 (9), 1891. 10.3390/ijms18091891 PMC5618540 28869494

[B37] Hait N. C. Allegood J. Maceyka M. Strub G. M. Harikumar K. B. Singh S. K. (2009). Regulation of Histone Acetylation in the Nucleus by Sphingosine-1-Phosphate. Science 325 (5945), 1254–1257. 10.1126/science.1176709 19729656 PMC2850596

[B38] Hait N. C. Avni D. Yamada A. Nagahashi M. Aoyagi T. Aoki H. (2015). The Phosphorylated Prodrug FTY720 Is a Histone Deacetylase Inhibitor that Reactivates ERα Expression and Enhances Hormonal Therapy for Breast Cancer. Oncogenesis 4 (6), e156. 10.1038/oncsis.2015.16 26053034 PMC4753524

[B39] Hamada M. Kameyama H. Iwai S. Yura Y. (2017). Induction of Autophagy by Sphingosine Kinase 1 Inhibitor PF-543 in Head and Neck Squamous Cell Carcinoma Cells. Cell Death Discov. 3 (1), 17047. 10.1038/cddiscovery.2017.47 29109864 PMC5554793

[B40] Hannun Y. A. Obeid L. M. (2018). Sphingolipids and Their Metabolism in Physiology and Disease. Nat. Rev. Mol. Cel Biol 19 (3), 175–191. 10.1038/nrm.2017.107 PMC5902181 29165427

[B41] Harada J. Foley M. Moskowitz M. A. Waeber C. (2004). Sphingosine-1-phosphate Induces Proliferation and Morphological Changes of Neural Progenitor Cells. J. Neurochem. 88 (4), 1026–1039. 10.1046/j.1471-4159.2003.02219.x 14756825

[B42] He X. H'ng S.-C. Leong D. T. Hutmacher D. W. Melendez A. J. (2010). Sphingosine-1-phosphate Mediates Proliferation Maintaining the Multipotency of Human Adult Bone Marrow and Adipose Tissue-Derived Stem Cells. J. Mol. Cel Biol. 2 (4), 199–208. 10.1093/jmcb/mjq011 20584786

[B43] Hii L.-W. Chung F. F.-L. Mai C. W. Yee Z. Y. Chan H. H. Raja V. J. (2020). Sphingosine Kinase 1 Regulates the Survival of Breast Cancer Stem Cells and Non-stem Breast Cancer Cells by Suppression of STAT1. Cells 9 (4), 886. 10.3390/cells9040886 PMC7226795 32260399

[B44] Hirata N. Yamada S. Shoda T. Kurihara M. Sekino Y. Kanda Y. (2014). Sphingosine-1-phosphate Promotes Expansion of Cancer Stem Cells via S1PR3 by a Ligand-independent Notch Activation. Nat. Commun. 5, 4806. 10.1038/ncomms5806 25254944

[B45] Inniss K. Moore H. (2006). Mediation of Apoptosis and Proliferation of Human Embryonic Stem Cells by Sphingosine-1-Phosphate. Stem Cell Dev. 15 (6), 789–796. 10.1089/scd.2006.15.789 17253942

[B46] Jarman K. E. Moretti P. A. B. Zebol J. R. Pitson S. M. (2010). Translocation of Sphingosine Kinase 1 to the Plasma Membrane Is Mediated by Calcium- and Integrin-Binding Protein 1. J. Biol. Chem. 285 (1), 483–492. 10.1074/jbc.M109.068395 19854831 PMC2804196

[B47] Ju T. Gao D. Fang Z.-Y. (2016). Targeting Colorectal Cancer Cells by a Novel Sphingosine Kinase 1 Inhibitor PF-543. Biochem. Biophysical Res. Commun. 470 (3), 728–734. 10.1016/j.bbrc.2016.01.053 26775841

[B48] Katsuta E. Yan L. Nagahashi M. Raza A. Sturgill J. L. Lyon D. E. (2017). Doxorubicin Effect Is Enhanced by Sphingosine-1-Phosphate Signaling Antagonist in Breast Cancer. J. Surg. Res. 219, 202–213. 10.1016/j.jss.2017.05.101 29078883 PMC5661979

[B49] Kawamori T. Kaneshiro T. Okumura M. Maalouf S. Uflacker A. Bielawski J. (2009). Role for Sphingosine Kinase 1 in colon Carcinogenesis. FASEB j. 23 (2), 405–414. 10.1096/fj.08-117572 18824518 PMC2630788

[B50] Kedderis L. B. Bozigian H. P. Kleeman J. M. Hall R. L. Palmer T. E. Harrison S. D. Jr (1995). Toxicity of the Protein Kinase C Inhibitor Safingol Administered Alone and in Combination with Chemotherapeutic Agents. Toxicol. Sci. 25 (2), 201–217. 10.1093/toxsci/25.2.201 7665004

[B51] Khodarev N. N. Roizman B. Weichselbaum R. R. (2012). Molecular Pathways: Interferon/Stat1 Pathway: Role in the Tumor Resistance to Genotoxic Stress and Aggressive Growth. Clin. Cancer Res. 18 (11), 3015–3021. 10.1158/1078-0432.Ccr-11-3225 22615451

[B52] Kim E.-S. Cha Y. Ham M. Jung J. Kim S. G. Hwang S. (2014). Inflammatory Lipid Sphingosine-1-Phosphate Upregulates C-Reactive Protein via C/EBPβ and Potentiates Breast Cancer Progression. Oncogene 33 (27), 3583–3593. 10.1038/onc.2013.319 23955082

[B53] Kim E.-S. Kim J.-S. Kim S. G. Hwang S. Lee C. H. Moon A. (2011). Sphingosine 1-phosphate Regulates Matrix Metalloproteinase-9 Expression and Breast Cell Invasion through S1P3-Gαq Coupling. J. Cel Sci 124 (13), 2220–2230. 10.1242/jcs.076794 21652634

[B54] Kohno M. Momoi M. Oo M. L. Paik J.-H. Lee Y.-M. Venkataraman K. (2006). Intracellular Role for Sphingosine Kinase 1 in Intestinal Adenoma Cell Proliferation. Mol. Cel Biol 26 (19), 7211–7223. 10.1128/mcb.02341-05 PMC1592880 16980623

[B55] Kotelevets N. Fabbro D. Huwiler A. Zangemeister-Wittke U. (2012). Targeting Sphingosine Kinase 1 in Carcinoma Cells Decreases Proliferation and Survival by Compromising PKC Activity and Cytokinesis. PLoS One 7 (6), e39209. 10.1371/journal.pone.0039209 22761740 PMC3382615

[B56] Kunkel G. T. Maceyka M. Milstien S. Spiegel S. (2013). Targeting the Sphingosine-1-Phosphate axis in Cancer, Inflammation and beyond. Nat. Rev. Drug Discov. 12 (9), 688–702. 10.1038/nrd4099 23954895 PMC3908769

[B57] Leclercq T. M. Moretti P. A. B. Vadas M. A. Pitson S. M. (2008). Eukaryotic Elongation Factor 1A Interacts with Sphingosine Kinase and Directly Enhances its Catalytic Activity. J. Biol. Chem. 283 (15), 9606–9614. 10.1074/jbc.m708782200 18263879 PMC2442288

[B58] Lee H. Deng J. Kujawski M. Yang C. Liu Y. Herrmann A. (2010). STAT3-induced S1PR1 Expression Is Crucial for Persistent STAT3 Activation in Tumors. Nat. Med. 16 (12), 1421–1428. 10.1038/nm.2250 21102457 PMC3088498

[B59] Legrier M.-E. Bièche I. Gaston J. Beurdeley A. Yvonnet V. Déas O. (2016). Activation of IFN/STAT1 Signalling Predicts Response to Chemotherapy in Oestrogen Receptor-Negative Breast Cancer. Br. J. Cancer 114 (2), 177–187. 10.1038/bjc.2015.398 26695443 PMC4815803

[B60] Li W. Yu C.-P. Xia J.-t. Zhang L. Weng G.-X. Zheng H.-q. (2009). Sphingosine Kinase 1 Is Associated with Gastric Cancer Progression and Poor Survival of Patients. Clin. Cancer Res. 15 (4), 1393–1399. 10.1158/1078-0432.Ccr-08-1158 19228740

[B61] Liang J. Nagahashi M. Kim E. Y. Harikumar K. B. Yamada A. Huang W.-C. (2013). Sphingosine-1-phosphate Links Persistent STAT3 Activation, Chronic Intestinal Inflammation, and Development of Colitis-Associated Cancer. Cancer Cell 23 (1), 107–120. 10.1016/j.ccr.2012.11.013 23273921 PMC3578577

[B62] Lim K. G. Tonelli F. Li Z. Lu X. Bittman R. Pyne S. (2011). FTY720 Analogues as Sphingosine Kinase 1 Inhibitors. J. Biol. Chem. 286 (21), 18633–18640. 10.1074/jbc.m111.220756 21464128 PMC3099679

[B63] Ling L.-U. Tan K.-B. Lin H. Chiu G. N. C. (2011). The Role of Reactive Oxygen Species and Autophagy in Safingol-Induced Cell Death. Cell Death Dis 2 (3), e129. 10.1038/cddis.2011.12 21390063 PMC3101809

[B64] Ling L. U. Lin H. Tan K. B. Chiu G. N. (2009). The Role of Protein Kinase C in the Synergistic Interaction of Safingol and Irinotecan in colon Cancer Cells. Int. J. Oncol. 35 (6), 1463–1471. 10.3892/ijo_00000465 19885570

[B65] Liu H. Sugiura M. Nava V. E. Edsall L. C. Kono K. Poulton S. (2000). Molecular Cloning and Functional Characterization of a Novel Mammalian Sphingosine Kinase Type 2 Isoform. J. Biol. Chem. 275 (26), 19513–19520. 10.1074/jbc.m002759200 10751414

[B66] Liu Y. Deng J. Wang L. Lee H. Armstrong B. Scuto A. (2012). S1PR1 Is an Effective Target to Block STAT3 Signaling in Activated B Cell-like Diffuse Large B-Cell Lymphoma. Blood J. Am. Soc. Hematol. 120 (7), 1458–1465. 10.1182/blood-2011-12-399030 PMC3423784 22745305

[B67] Long J. S. Edwards J. Watson C. Tovey S. Mair K. M. Schiff R. (2010). Sphingosine Kinase 1 Induces Tolerance to Human Epidermal Growth Factor Receptor 2 and Prevents Formation of a Migratory Phenotype in Response to Sphingosine 1-phosphate in Estrogen Receptor-Positive Breast Cancer Cells. Mol. Cel Biol 30 (15), 3827–3841. 10.1128/MCB.01133-09 PMC2916408 20516217

[B68] Long J. S. Fujiwara Y. Edwards J. Tannahill C. L. Tigyi G. Pyne S. (2010). Sphingosine 1-phosphate Receptor 4 Uses HER2 (ERBB2) to Regulate Extracellular Signal Regulated Kinase-1/2 in MDA-MB-453 Breast Cancer Cells. J. Biol. Chem. 285 (46), 35957–35966. 10.1074/jbc.m110.117945 20837468 PMC2975218

[B69] Loveridge C. Tonelli F. Leclercq T. Lim K. G. Long J. S. Berdyshev E. (2010). The Sphingosine Kinase 1 Inhibitor 2-(p-Hydroxyanilino)-4-(p-Chlorophenyl)thiazole Induces Proteasomal Degradation of Sphingosine Kinase 1 in Mammalian Cells. J. Biol. Chem. 285 (50), 38841–38852. 10.1074/jbc.m110.127993 20926375 PMC2998105

[B70] Lynch K. R. (2012). Building a Better Sphingosine Kinase-1 Inhibitor. Biochem. J. 444 (1), e1–e2. 10.1042/bj20120567 22533672

[B71] Ma Y. Xing X. Kong R. Cheng C. Li S. Yang X. (2021). SphK1 Promotes Development of Non-small C-ell L-ung C-ancer through A-ctivation of STAT3. Int. J. Mol. Med. 47 (1), 374–386. 10.3892/ijmm.2020.4796 33236138

[B72] Maceyka M. Nava V. E. Milstien S. Spiegel S. (2004). Aminoacylase 1 Is a Sphingosine Kinase 1-interacting Protein. FEBS Lett. 568 (1-3), 30–34. 10.1016/j.febslet.2004.04.093 15196915

[B73] Maczis M. A. Maceyka M. Waters M. R. Newton J. Singh M. Rigsby M. F. (2018). Sphingosine Kinase 1 Activation by Estrogen Receptor α36 Contributes to Tamoxifen Resistance in Breast Cancer. J. Lipid Res. 59 (12), 2297–2307. 10.1194/jlr.m085191 30315000 PMC6277156

[B74] Maczis M. Milstien S. Spiegel S. (2016). Sphingosine-1-phosphate and Estrogen Signaling in Breast Cancer. Adv. Biol. Regul. 60, 160–165. 10.1016/j.jbior.2015.09.006 26601898

[B75] Maiti A. Takabe K. Hait N. C. (2017). Metastatic Triple-Negative Breast Cancer Is Dependent on SphKs/S1P Signaling for Growth and Survival. Cell Signal. 32, 85–92. 10.1016/j.cellsig.2017.01.021 28108260 PMC5731460

[B76] Marotta L. L. C. Almendro V. Marusyk A. Shipitsin M. Schemme J. Walker S. R. (2011). The JAK2/STAT3 Signaling Pathway Is Required for Growth of CD44+CD24- Stem Cell-like Breast Cancer Cells in Human Tumors. J. Clin. Invest. 121 (7), 2723–2735. 10.1172/jci44745 21633165 PMC3223826

[B77] Martin J. L. de Silva H. C. Lin M. Z. Scott C. D. Baxter R. C. (2014). Inhibition of Insulin-like Growth Factor-Binding Protein-3 Signaling through Sphingosine Kinase-1 Sensitizes Triple-Negative Breast Cancer Cells to EGF Receptor Blockade. Mol. Cancer Ther. 13 (2), 316–328. 10.1158/1535-7163.mct-13-0367 24337110

[B78] Nagahashi M. Yamada A. Katsuta E. Aoyagi T. Huang W.-C. Terracina K. P. (2018). Targeting the SphK1/S1P/S1PR1 axis that Links Obesity, Chronic Inflammation, and Breast Cancer Metastasis. Cancer Res. 78 (7), 1713–1725. 10.1158/0008-5472.can-17-1423 29351902 PMC6945803

[B79] Nava V. Hobson J. P. Murthy S. Milstien S. Spiegel S. (2002). Sphingosine Kinase Type 1 Promotes Estrogen-dependent Tumorigenesis of Breast Cancer MCF-7 Cells. Exp. Cel Res. 281 (1), 115–127. 10.1006/excr.2002.5658 12441135

[B80] Ogretmen B. (2018). Sphingolipid Metabolism in Cancer Signalling and Therapy. Nat. Rev. Cancer 18 (1), 33–50. 10.1038/nrc.2017.96 29147025 PMC5818153

[B81] Ohotski J. Edwards J. Elsberger B. Watson C. Orange C. Mallon E. (2013). Identification of Novel Functional and Spatial Associations between Sphingosine Kinase 1, Sphingosine 1-phosphate Receptors and Other Signaling Proteins that Affect Prognostic Outcome in Estrogen Receptor-Positive Breast Cancer. Int. J. Cancer 132 (3), 605–616. 10.1002/ijc.27692 22733311

[B82] Ohotski J. Long J. S. Orange C. Elsberger B. Mallon E. Doughty J. (2012). Expression of Sphingosine 1-phosphate Receptor 4 and Sphingosine Kinase 1 Is Associated with Outcome in Oestrogen Receptor-Negative Breast Cancer. Br. J. Cancer 106 (8), 1453–1459. 10.1038/bjc.2012.98 22460268 PMC3326679

[B83] Ohotski J. Rosen H. Bittman R. Pyne S. Pyne N. J. (2014). Sphingosine Kinase 2 Prevents the Nuclear Translocation of Sphingosine 1-phosphate Receptor-2 and Tyrosine 416 Phosphorylated C-Src and Increases Estrogen Receptor Negative MDA-MB-231 Breast Cancer Cell Growth: the Role of Sphingosine 1-phosphate Receptor-4. Cell Signal. 26 (5), 1040–1047. 10.1016/j.cellsig.2014.01.023 24486401

[B84] Olivera A. Kohama T. Tu Z. Milstien S. Spiegel S. (1998). Purification and Characterization of Rat Kidney Sphingosine Kinase. J. Biol. Chem. 273 (20), 12576–12583. 10.1074/jbc.273.20.12576 9575218

[B85] Panneer Selvam S. De Palma R. M. Oaks J. J. Oleinik N. Peterson Y. K. Stahelin R. V. (2015). Binding of the Sphingolipid S1P to hTERT Stabilizes Telomerase at the Nuclear Periphery by Allosterically Mimicking Protein Phosphorylation. Sci. Signal. 8 (381), ra58–ra. 10.1126/scisignal.aaa4998 26082434 PMC4492107

[B86] Pébay A. Bonder C. S. Pitson S. M. (2007). Stem Cell Regulation by Lysophospholipids. Prostaglandins Other Lipid Mediat 84 (3-4), 83–97. 10.1016/j.prostaglandins.2007.08.004 17991611

[B87] Pébay A. Wong R. C. B. Pitson S. M. Wolvetang E. J. Peh G. S.-L. Filipczyk A. (2005). Essential Roles of Sphingosine-1-Phosphate and Platelet-Derived Growth Factor in the Maintenance of Human Embryonic Stem Cells. Stem Cells 23 (10), 1541–1548. 10.1634/stemcells.2004-0338 16081668

[B88] Perrotti D. Neviani P. (2013). Protein Phosphatase 2A: a Target for Anticancer Therapy. Lancet Oncol. 14 (6), e229–e238. 10.1016/s1470-2045(12)70558-2 23639323 PMC3913484

[B89] Pinho F. G. Frampton A. E. Nunes J. Krell J. Alshaker H. Jacob J. (2013). Downregulation of microRNA-515-5p by the Estrogen Receptor Modulates Sphingosine Kinase 1 and Breast Cancer Cell Proliferation. Cancer Res. 73 (19), 5936–5948. 10.1158/0008-5472.can-13-0158 23928990

[B90] Pitson S. M. (2011). Regulation of Sphingosine Kinase and Sphingolipid Signaling. Trends Biochem. Sci. 36 (2), 97–107. 10.1016/j.tibs.2010.08.001 20870412

[B91] Pitson S. M. Xia P. Leclercq T. M. Moretti P. A. B. Zebol J. R. Lynn H. E. (2005). Phosphorylation-dependent Translocation of Sphingosine Kinase to the Plasma Membrane Drives its Oncogenic Signalling. J. Exp. Med. 201 (1), 49–54. 10.1084/jem.20040559 15623571 PMC2212769

[B92] Price S. T. Beckham T. H. Cheng J. C. Lu P. Liu X. Norris J. S. (2015). Sphingosine 1-phosphate Receptor 2 Regulates the Migration, Proliferation, and Differentiation of Mesenchymal Stem Cells. Int. J. Stem Cel Res Ther 2 (2), 014. 10.23937/2469-570x/1410014 PMC4888913 27275017

[B93] Pyne N. J. McNaughton M. Boomkamp S. MacRitchie N. Evangelisti C. Martelli A. M. (2016). Role of Sphingosine 1-phosphate Receptors, Sphingosine Kinases and Sphingosine in Cancer and Inflammation. Adv. Biol. Regul. 60, 151–159. 10.1016/j.jbior.2015.09.001 26429117

[B94] Pyne N. J. Pyne S. (2013). Sphingosine 1-phosphate Is a Missing Link between Chronic Inflammation and colon Cancer. Cancer Cell 23 (1), 5–7. 10.1016/j.ccr.2012.12.005 23328479

[B95] Pyne S. Adams D. R. Pyne N. J. (2016). Sphingosine 1-phosphate and Sphingosine Kinases in Health and Disease: Recent Advances. Prog. Lipid Res. 62, 93–106. 10.1016/j.plipres.2016.03.001 26970273

[B96] Pyne S. Edwards J. Ohotski J. Pyne N. J. (2012). Sphingosine 1-phosphate Receptors and Sphingosine Kinase 1: Novel Biomarkers for Clinical Prognosis in Breast, Prostate, and Hematological Cancers. Front. Oncol. 2, 168. 10.3389/fonc.2012.00168 23316473 PMC3540928

[B97] Quinn P. Wang X. (2008). Lipids in Health and Disease. Springer Netherlands.

[B98] Ren S. Xin C. Pfeilschifter J. Huwiler A. (2010). A Novel Mode of Action of the Putative Sphingosine Kinase Inhibitor 2-(p-Hydroxyanilino)-4-(p-Chlorophenyl) Thiazole (SKI II): Induction of Lysosomal Sphingosine Kinase 1 Degradation. Cell Physiol Biochem 26 (1), 97–104. 10.1159/000315110 20502009

[B99] Rincón R. Cristóbal I. Zazo S. Arpí O. Menéndez S. Manso R. (2015). PP2A Inhibition Determines Poor Outcome and Doxorubicin Resistance in Early Breast Cancer and its Activation Shows Promising Therapeutic Effects. Oncotarget 6 (6), 4299–4314. 10.18632/oncotarget.3012 25726524 PMC4414191

[B100] Rodgers A. Mormeneo D. Long J. S. Delgado A. Pyne N. J. Pyne S. (2009). Sphingosine 1-phosphate Regulation of Extracellular Signal-Regulated Kinase-1/2 in Embryonic Stem Cells. Stem Cell Dev. 18 (9), 1319–1330. 10.1089/scd.2009.0023 19228106

[B101] Ryu J. M. Baek Y. B. Shin M. S. Park J. H. Park S. H. Lee J. H. (2014). Sphingosine-1-phosphate-induced Flk-1 Transactivation Stimulates Mouse Embryonic Stem Cell Proliferation through S1P1/S1P3-dependent β-arrestin/c-Src Pathways. Stem Cel Res. 12 (1), 69–85. 10.1016/j.scr.2013.08.013 24145189

[B102] Saddoughi S. A. Gencer S. Peterson Y. K. Ward K. E. Mukhopadhyay A. Oaks J. (2013). Sphingosine Analogue Drug FTY720 Targets I2PP2A/SET and Mediates Lung Tumour Suppression via Activation of PP2A‐RIPK1‐dependent Necroptosis. EMBO Mol. Med. 5 (1), 105–121. 10.1002/emmm.201201283 23180565 PMC3569657

[B103] Santos W. L. Lynch K. R. (2015). Drugging Sphingosine Kinases. ACS Chem. Biol. 10 (1), 225–233. 10.1021/cb5008426 25384187 PMC4301069

[B104] Sarkar S. Maceyka M. Hait N. C. Paugh S. W. Sankala H. Milstien S. (2005). Sphingosine Kinase 1 Is Required for Migration, Proliferation and Survival of MCF-7 Human Breast Cancer Cells. FEBS Lett. 579 (24), 5313–5317. 10.1016/j.febslet.2005.08.055 16194537

[B105] Schnute M. E. McReynolds M. D. Kasten T. Yates M. Jerome G. Rains J. W. (2012). Modulation of Cellular S1P Levels with a Novel, Potent and Specific Inhibitor of Sphingosine Kinase-1. Biochem. J. 444 (1), 79–88. 10.1042/bj20111929 22397330

[B106] Schwartz G. K. Ward D. Saltz L. Casper E. S. Spiess T. Mullen E. (1997). A Pilot Clinical/pharmacological Study of the Protein Kinase C-specific Inhibitor Safingol Alone and in Combination with Doxorubicin. Clin. Cancer Res. 3 (4), 537–543. 9815717

[B107] Schwartz G. K. Haimovitz-Friedman A. Dhupar S. K. Ehleiter D. Maslak P. Lai L. (1995). Potentiation of Apoptosis by Treatment with the Protein Kinase C-specific Inhibitor Safingol in Mitomycin C-Treated Gastric Cancer Cells. JNCI J. Natl. Cancer Inst. 87 (18), 1394–1399. 10.1093/jnci/87.18.1394 7658500

[B108] Snider A. J. Kawamori T. Bradshaw S. G. Orr K. A. Gilkeson G. S. Hannun Y. A. (2009). A Role for Sphingosine Kinase 1 in Dextran Sulfate Sodium‐induced Colitis. FASEB j. 23 (1), 143–152. 10.1096/fj.08-118109 18815359 PMC2626622

[B109] Song L. Xiong H. Li J. Liao W. Wang L. Wu J. (2011). Sphingosine Kinase-1 Enhances Resistance to Apoptosis through Activation of PI3K/Akt/NF-Κb Pathway in Human Non-small Cell Lung Cancer. Clin. Cancer Res. 17 (7), 1839–1849. 10.1158/1078-0432.CCR-10-0720 21325072

[B110] Strader C. R. Pearce C. J. Oberlies N. H. (2011). Fingolimod (FTY720): a Recently Approved Multiple Sclerosis Drug Based on a Fungal Secondary Metabolite. J. Nat. Prod. 74 (4), 900–907. 10.1021/np2000528 21456524

[B111] Strub G. M. Maceyka M. Hait N. C. Milstien S. Spiegel S. (2010). Extracellular and Intracellular Actions of Sphingosine-1-Phosphate. in Sphingolipids as Signaling and Regulatory Molecules. Springer, 141–155. 10.1007/978-1-4419-6741-1_10 PMC2951632 20919652

[B112] Strub G. M. Paillard M. Liang J. Gomez L. Allegood J. C. Hait N. C. (2011). Sphingosine‐1‐phosphate Produced by Sphingosine Kinase 2 in Mitochondria Interacts with Prohibitin 2 to Regulate Complex IV Assembly and Respiration. FASEB j. 25 (2), 600–612. 10.1096/fj.10-167502 20959514 PMC3023391

[B113] Sukocheva O. A. Hu D. G. Meech R. Bishayee A. (2021). Divergence of Intracellular Trafficking of Sphingosine Kinase 1 and Sphingosine-1-Phosphate Receptor 3 in MCF-7 Breast Cancer Cells and MCF-7-Derived Stem Cell-Enriched Mammospheres. Ijms 22 (9), 4314. 10.3390/ijms22094314 33919234 PMC8122545

[B114] Sukocheva O. A. Wang L. Albanese N. Pitson S. M. Vadas M. A. Xia P. (2003). Sphingosine Kinase Transmits Estrogen Signaling in Human Breast Cancer Cells. Mol. Endocrinol. 17 (10), 2002–2012. 10.1210/me.2003-0119 12881510

[B115] Sukocheva O. Wadham C. (2014). Role of Sphingolipids in Oestrogen Signalling in Breast Cancer Cells: an Update. J. Endocrinol. 220 (3), R25–R35. 10.1530/joe-13-0388 24323911

[B116] Sukocheva O. Wadham C. Holmes A. Albanese N. Verrier E. Feng F. (2006). Estrogen Transactivates EGFR via the Sphingosine 1-phosphate Receptor Edg-3: the Role of Sphingosine Kinase-1. J. Cel. Biol. 173 (2), 301–310. 10.1083/jcb.200506033 PMC2063820 16636149

[B117] Taha T. A. Kitatani K. El‐Alwani M. Bielawski J. Hannun Y. A. Obeid L. M. (2006). Loss of Sphingosine Kinase‐1 Activates the Intrinsic Pathway of Programmed Cell Death: Modulation of Sphingolipid Levels and the Induction of Apoptosis. FASEB j. 20 (3), 482–484. 10.1096/fj.05-4412fje 16507765

[B118] Taha T. A. Osta W. Kozhaya L. Bielawski J. Johnson K. R. Gillanders W. E. (2004). Down-regulation of Sphingosine Kinase-1 by DNA Damage. J. Biol. Chem. 279 (19), 20546–20554. 10.1074/jbc.M401259200 14988393

[B119] Tan S. S. L. Khin L. W. Wong L. Yan B. Ong C. W. Datta A. (2014). Sphingosine Kinase 1 Promotes Malignant Progression in colon Cancer and Independently Predicts Survival of Patients with colon Cancer by Competing Risk Approach in South Asian Population. Clin. Translational Gastroenterol. 5 (2), e51. 10.1038/ctg.2013.21 PMC3940836 24572701

[B120] Thudichum J. (1884). Treatise on the Chemical Constitution of the Brain, 1962. London: Bailliere, Tindall and Cox, 1–262.

[B121] Tonelli F. Lim K. G. Loveridge C. Long J. Pitson S. M. Tigyi G. (2010). FTY720 and (S)-FTY720 Vinylphosphonate Inhibit Sphingosine Kinase 1 and Promote its Proteasomal Degradation in Human Pulmonary Artery Smooth Muscle, Breast Cancer and Androgen-independent Prostate Cancer Cells. Cell Signal. 22 (10), 1536–1542. 10.1016/j.cellsig.2010.05.022 20570726 PMC2947314

[B122] Vadas M. Xia P. McCaughan G. Gamble J. (2008). The Role of Sphingosine Kinase 1 in Cancer: Oncogene or Non-oncogene Addiction? Biochim. Biophys. Acta (Bba) - Mol. Cel Biol. Lipids 1781 (9), 442–447. 10.1016/j.bbalip.2008.06.007 18638570

[B123] Wang S. Liang Y. Chang W. Hu B. Zhang Y. (2018). Triple Negative Breast Cancer Depends on Sphingosine Kinase 1 (SphK1)/sphingosine-1-Phosphate (S1P)/sphingosine 1-phosphate Receptor 3 (S1PR3)/Notch Signaling for Metastasis. Med. Sci. Monit. 24, 1912–1923. 10.12659/msm.905833 29605826 PMC5894569

[B124] Wang Y.-C. Tsai C.-F. Chuang H.-L. Chang Y.-C. Chen H.-S. Lee J.-N. (2016). Benzyl Butyl Phthalate Promotes Breast Cancer Stem Cell Expansion via SPHK1/S1P/S1PR3 Signaling. Oncotarget 7 (20), 29563–29576. 10.18632/oncotarget.9007 27129165 PMC5045417

[B125] Watson C. Long J. S. Orange C. Tannahill C. L. Mallon E. McGlynn L. M. (2010). High Expression of Sphingosine 1-phosphate Receptors, S1P1 and S1P3, Sphingosine Kinase 1, and Extracellular Signal-Regulated Kinase-1/2 Is Associated with Development of Tamoxifen Resistance in Estrogen Receptor-Positive Breast Cancer Patients. Am. J. Pathol. 177 (5), 2205–2215. 10.2353/ajpath.2010.100220 20889557 PMC2966780

[B126] Watson D. G. Tonelli F. Alossaimi M. Williamson L. Chan E. Gorshkova I. (2013). The Roles of Sphingosine Kinases 1 and 2 in Regulating the Warburg Effect in Prostate Cancer Cells. Cell Signal. 25 (4), 1011–1017. 10.1016/j.cellsig.2013.01.002 23314175 PMC3595369

[B127] White C. Alshaker H. Cooper C. Winkler M. Pchejetski D. (2016). The Emerging Role of FTY720 (Fingolimod) in Cancer Treatment. Oncotarget 7 (17), 23106–23127. 10.18632/oncotarget.7145 27036015 PMC5029614

[B128] Wong R. C. B. Tellis I. Jamshidi P. Pera M. Pébay A. (2007). Anti-apoptotic Effect of Sphingosine-1-Phosphate and Platelet-Derived Growth Factor in Human Embryonic Stem Cells. Stem Cell Dev. 16 (6), 989–1002. 10.1089/scd.2007.0057 18047416

[B129] Woo S. M. Seo B. R. Min K.-j. Kwon T. K. (2015). FTY720 Enhances TRAIL-Mediated Apoptosis by Up-Regulating DR5 and Down-Regulating Mcl-1 in Cancer Cells. Oncotarget 6 (13), 11614–11626. 10.18632/oncotarget.3426 25843953 PMC4484480

[B130] Xia P. Wang L. Moretti P. A. B. Albanese N. Chai F. Pitson S. M. (2002). Sphingosine Kinase Interacts with TRAF2 and Dissects Tumor Necrosis Factor-α Signaling. J. Biol. Chem. 277 (10), 7996–8003. 10.1074/jbc.m111423200 11777919

[B131] Xiong H. Wang J. Guan H. Wu J. Xu R. Wang M. (2014). SphK1 Confers Resistance to Apoptosis in Gastric Cancer Cells by Downregulating Bim via Stimulating Akt/FoxO3a Signaling. Oncol. Rep. 32 (4), 1369–1373. 10.3892/or.2014.3391 25109605 PMC4148362

[B132] Yokota S. Taniguchi Y. Kihara A. Mitsutake S. Igarashi Y. (2004). Asp177 in C4 Domain of Mouse Sphingosine Kinase 1a Is Important for the Sphingosine Recognition. FEBS Lett. 578 (1-2), 106–110. 10.1016/j.febslet.2004.10.081 15581625

[B133] Yu M. Zhang K. Wang S. Xue L. Chen Z. Feng N. (2021). Increased SPHK1 and HAS2 Expressions Correlate to Poor Prognosis in Pancreatic Cancer. Biomed. Res. Int. 2021, 1–8. 10.1155/2021/8861766 PMC7806397 33506044

[B134] Zhang L. Wang H.-D. Ji X.-J. Cong Z.-X. Zhu J.-H. Zhou Y. (2013). FTY720 for Cancer Therapy (Review). Oncol. Rep. 30 (6), 2571–2578. 10.3892/or.2013.2765 24100923

[B135] Zhang Y. Wang Y. Wan Z. Liu S. Cao Y. Zeng Z. (2014). Sphingosine Kinase 1 and Cancer: a Systematic Review and Meta-Analysis. PLoS One 9 (2), e90362. 10.1371/journal.pone.0090362 24587339 PMC3937388

[B136] Zhu L. Wang Z. Lin Y. Chen Z. Liu H. Chen Y. (2015). Sphingosine Kinase 1 Enhances the Invasion and Migration of Non-small Cell Lung Cancer Cells via the AKT Pathway. Oncol. Rep. 33 (3), 1257–1263. 10.3892/or.2014.3683 25529771

[B137] Zhu W. Gliddon B. L. Jarman K. E. Moretti P. A. B. Tin T. Parise L. V. (2017). CIB1 Contributes to Oncogenic Signalling by Ras via Modulating the Subcellular Localisation of Sphingosine Kinase 1. Oncogene 36 (18), 2619–2627. 10.1038/onc.2016.428 27941888 PMC5418080

